# Efficacy of Oseltamivir-Zanamivir Combination Compared to Each Monotherapy for Seasonal Influenza: A Randomized Placebo-Controlled Trial

**DOI:** 10.1371/journal.pmed.1000362

**Published:** 2010-11-02

**Authors:** Xavier Duval, Sylvie van der Werf, Thierry Blanchon, Anne Mosnier, Maude Bouscambert-Duchamp, Annick Tibi, Vincent Enouf, Cécile Charlois-Ou, Corine Vincent, Laurent Andreoletti, Florence Tubach, Bruno Lina, France Mentré, Catherine Leport, and the Bivir Study Group

**Affiliations:** 1 Inserm CIC 007, APHP, Hôpital Bichat, Paris, France; 2 Inserm U738, Paris, France; 3 Université Paris Diderot, Paris 7, UFR de Médecine, site Bichat, Paris, France; 4 Institut Pasteur, Centre National de Référence des virus influenzae (Région-Nord), Unité de Génétique Moléculaire des Virus à ARN, Paris, France; 5 CNRS URA3015, Paris, France; 6 Université Paris Diderot, Paris 7, UFR Sciences du Vivant, Paris, France; 7 Inserm UPMC UMR-S 707, Faculté de médecine Pierre et Marie Curie, Paris, France; 8 Université Pierre et Marie Curie, Paris 6, UFR de Médecine, U707, Paris, France; 9 Réseau des Groupes Régionaux d'Observation de la Grippe (GROG), Coordination nationale, Paris, France; 10 Hospices Civils de Lyon, Centre National de Référence des virus influenzae (Région-Sud), GHE, Bron, France; 11 Université Lyon 1, VirPatH, CNRS FRE 3011, Lyon, France; 12 APHP- Agence Générale des Equipements et Produits de Santé, Unité Essais Cliniques, Paris, France; 13 Université Paris Descartes, Paris 5, Faculté de Pharmacie, Paris, France; 14 Université Paris Diderot, Paris 7, UFR de Médecine, site Bichat, Laboratoire de Recherche en Pathologie Infectieuse, Paris, France; 15 APHP, Hôpital Bichat, Unité de Biostatistiques, Paris, France; 16 Hôpital Robert Debré, Unité de Virologie médicale, Reims, France; 17 Unité de Virologie Médicale et Moléculaire Faculté de Médecine Université Champagne-Ardenne IFR53/EA-4303, Reims, France; 18 APHP Hôpital Bichat, Département d'Epidémiologie, Biostatistiques et Recherche Clinique, Paris, France; 19 APHP, Unité de Coordination des Risques Epidémiques et Biologiques, Paris, France; The University of Hong Kong, Hong Kong

## Abstract

**Background:**

Neuraminidase inhibitors are thought to be efficacious in reducing the time to alleviation of symptoms in outpatients with seasonal influenza. The objective of this study was to compare the short-term virological efficacy of oseltamivir-zanamivir combination versus each monotherapy plus placebo.

**Methods and Findings:**

We conducted a randomized placebo-controlled trial with 145 general practitioners throughout France during the 2008–2009 seasonal influenza epidemic. Patients, general practitioners, and outcome assessors were all blinded to treatment assignment. Adult outpatients presenting influenza-like illness for less than 36 hours and a positive influenza A rapid test diagnosis were randomized to oseltamivir 75 mg orally twice daily plus zanamivir 10 mg by inhalation twice daily (OZ), oseltamivir plus inhaled placebo (O), or zanamivir plus oral placebo (Z). Treatment efficacy was assessed virologically according to the proportion of patients with nasal influenza reverse transcription (RT)-PCR below 200 copies genome equivalent (cgeq)/µl at day 2 (primary outcome), and clinically to the time to alleviation of symptoms until day 14. Overall 541 patients (of the 900 planned) were included (OZ, *n* = 192; O, *n* = 176; Z, *n* = 173), 49% male, mean age 39 years. In the intention-to-treat analysis conducted in the 447 patients with RT-PCR-confirmed influenza A, 46%, 59%, and 34% in OZ (*n* = 157), O (*n* = 141), and Z (*n* = 149) arms had RT-PCR<200 cgeq/µl (−13.0%, 95% confidence interval [CI] −23.1 to −2.9, *p* = 0.025; +12.3%, 95% CI 2.39–22.2, *p* = 0.028 for OZ/O and OZ/Z comparisons). Mean day 0 to day 2 viral load decrease was 2.14, 2.49, and 1.68 log_10_ cgeq/µl (*p* = 0.060, *p* = 0.016 for OZ/O and OZ/Z). Median time to alleviation of symptoms was 4.0, 3.0, and 4.0 days (+1.0, 95% CI 0.0–4.0, *p* = 0.018; +0.0, 95% CI −3.0 to 3.0, *p* = 0.960 for OZ/O and OZ/Z). Four severe adverse events were observed. Nausea and/or vomiting tended to be more frequent in the combination arm (OZ, *n* = 13; O, *n* = 4; and Z, *n* = 5 patients, respectively).

**Conclusions:**

In adults with seasonal influenza A mainly H3N2 virus infection, the oseltamivir-zanamivir combination appeared less effective than oseltamivir monotherapy, and not significantly more effective than zanamivir monotherapy. Despite the theoretical potential for the reduction of the emergence of antiviral resistance, the lower effectiveness of this combination calls for caution in its use in clinical practice.

**Trial registration:**

www.ClinicalTrials.gov NCT00799760

*Please see later in the article for the Editors' Summary*

## Abstract 2

Analysis of virological and clinical outcomes from a randomized trial that was terminated early suggest that combined treatment of seasonal influenza in adult outpatients with oseltamivir plus zanamivir is no more effective than either oseltamivir or zanamivir monotherapy.

## Editors' Summary

**Background:**

In the last few years, use of the neuraminidase inhibitors, oseltamivir and zanamivir, has been considered a key strategy for limiting the impact of influenza both in individuals (by reducing morbidity and mortality) and collectively (by slowing the virus' spread to buy time for vaccine production, the cornerstone of influenza control). However, there are concerns that widespread use of a single antiviral drug may lead to resistant strains, which could dramatically reduce its effectiveness in future. Theoretically, if well tolerated, and if producing at least additive antiviral activity, the combination of two antiviral agents could offer several advantages such as reducing disease severity and reducing the viral shedding period, which in turn could lead to lower infection rates and reduced resistance especially in immunocompromised patients. Importantly, combining two drugs could ensure optimal treatment of all types of circulating influenza virus and subtypes or variants. The combination of two neuraminidase inhibitors is feasible as both oseltamivir and zanamivir are licensed for seasonal influenza and have different key mutations associated with resistance to each drug.

**Why Was This Study Done?:**

As yet, there have been no robust randomized controlled trials that compare the effectiveness of monotherapy with either oseltamivir or zanamivir with the effectiveness of a oseltamivir-zanamivir combination. Such a study would be important for influenza pandemic planning.

**What Did the Researchers Do and Find?:**

The researchers conducted a randomized, placebo-controlled trial within 145 general practitioners throughout France during the seasonal influenza epidemic in 2008–2009. Adults who visited their general practitioner with symptoms of an influenza-like illness for less than 36 hours and who had a positive influenza A rapid test were randomized to one of three arms: (1) oral oseltamivir 75 mg twice daily plus zanamivir 10 mg by inhalation twice daily, (2) oral oseltamivir 75 mg twice daily plus inhaled placebo, or (3) zanamivir 10 mg by inhalation twice daily plus oral placebo. The effects of the drugs or combination of drugs was assessed virologically, by looking at the proportion of patients with nasal influenza reverse transcription (RT)-PCR below a particular level on day 2 of treatment. Clinical measures of effectiveness included the time to resolution of illness, the number of patients with alleviation of symptoms at the end of treatment, and the incidence of secondary complications of influenza such as otitis, bronchitis, sinusitis, and pneumonia. In the study, patients, general practitioners, and outcome assessors were all blinded to treatment assignments. Due to the emergence of the H1N1 pandemic in 2009, the study's independent data-monitoring committee requested that the researchers terminate the trial early and analyze the results earlier than planned.

541 patients (of the 900 planned) were enrolled in the study (192 in group 1; 176 in group 2; and 173 in group 3) of whom 447 were infected with influenza A. Overall the oseltamivir-zanamivir combination was both virologically and clinically significantly less effective than the oseltamivir monotherapy. In addition, the clinical effects of the oseltamivir-zanamivir combination on time to resolution of symptoms were not significantly different from that of zanamivir monotherapy, suggesting that oseltamivir does not add clinical benefit to zanamivir monotherapy.

**What Do These Findings Mean?:**

The results of this study essentially show that in France during the Winter of 2009 prepandemic (of which 85% was due to of H3N2 virus), in adults with seasonal influenza A virus infection, the combination of oseltamivir and zanamivir was less effective than oseltamivir monotherapy and not significantly more effective than zanamivir monotherapy. These results call for caution in the use of the oseltamivir-zanamivir combination in treatment of adult outpatients. In addition, as the clinical and virological effects of oseltamivir monotherapy over zanamivir monotherapy were superior in this trial, oseltamivir should be the recommended treatment during influenza seasons with predominant H3N2 viruses. However, the results of this study should be confirmed in the coming years on future circulating influenza viruses.

**Additional Information:**

Please access these Web sites via the online version of this summary at http://dx.doi.org/10.1371/journal.pmed.1000362.

- Wikipedia has information on H3N3 influenza A virus (note that Wikipedia is a free online encyclopedia that anyone can edit; available in several languages)
- The World Health Organization has a global alert and response site on seasonal influenza
- Patient UK provides information about antivirals for influenza
- Answers.com has information about oseltamivir and about zanamivir

## Introduction

Neuraminidase inhibitors (oseltamivir [O], zanamivir [Z]) are thought to be efficacious as compared to placebo in outpatients with uncomplicated seasonal influenza [1]–[6], both clinically in terms of reduction in duration of symptoms, as well as in terms of a reduction in viral shedding. In 2008, they were considered an important strategy to limit the impact of an influenza pandemic both individually, by reducing morbidity and mortality, and collectively, by slowing spread of the virus to allow time for vaccine production, the cornerstone of influenza control [2]–[4],[7]. It was hypothesized that the widespread use of a single antiviral might result in the emergence of resistant strains whose subsequent spread could dramatically reduce the effectiveness of antiviral therapy. The combination of two antiviral agents, if well tolerated, and if producing at least additive antiviral activity, theoretically offers several advantages: reducing disease severity, viral shedding, and viral excretion period, thereby also lowering the attack rate and risk of selection of resistant viruses, specifically in individuals with prolonged viral shedding, such as immunocompromised patients [8],[9]. Indeed, mathematical modelling showed a reduction in risk of emergence of resistant strains during early phases of a pandemic, associated with use of two antivirals as compared to single antiviral therapy [9]. Finally, another theoretical advantage of combining two drugs would be to ensure optimal treatment of all circulating influenza virus types, subtypes, or variants, as susceptibility of influenza viruses has been shown to vary, and seasonal H1N1 viruses naturally resistant to oseltamivir, which remain susceptible to zanamivir, emerged in 2008 [10]. Among antivirals active against influenza virus, the combination of neuraminidase inhibitors is attractive, because both compounds are licensed for seasonal influenza, they are delivered to the respiratory tract by distinct means (directly through a diskhaler for zanamivir, after gastrointestinal absorption and hepatic metabolism for oseltamivir), and key mutations associated with resistance are different for each drug. However, negative interactions cannot not be ruled out owing to the possible competition between these two drugs, which target the same binding pocket in the neuraminidase.

In 2006, in the context of pandemic planning, we designed a double-placebo randomized controlled trial in patients presenting with seasonal influenza-like illness to compare the oseltamivir-zanamivir combination to each of the monotherapies plus placebo. The trial was conducted in France, during the winter of 2008–2009. Because of the emergence of the pandemic 2009 (H1N1) virus in humans, in April 2009 in North America, and its subsequent worldwide spread, the independent data-monitoring committee requested that we terminate the trial early and analyze the results earlier than planned, given the possible impact of the results on antiviral treatment management during the pandemic [11].

## Methods

### Patients

From January 7th to March 15th 2009 (period of the winter 2008–2009 influenza epidemic in France), we enrolled throughout France adults 18 y old and older who consulted their general practitioner within 36 h of influenza symptoms onset (following the first influenza symptoms reported by the patient), with a temperature greater than or equal to 38°C (reported or observed by the practitioner), one or more respiratory symptoms (cough, sore throat), one or more general symptoms (headache, dizziness, myalgia, sweats and or chills, fatigue), and a positive nasal rapid test for influenza A (Clearview Exact Influenza A & B) performed by the practitioner. Enrolment of women required a negative urine pregnancy test. Exclusion criteria were vaccination against influenza during the 2008–2009 season, recent exacerbations of chronic obstructive pulmonary disease (COPD), asthma or severe chronic disease, previous history of depression, and prior inclusion in this trial. Prior to inclusion, patients gave informed written consent. The protocol was approved by the Ethics Committee of Ile de France 1 (Texts S1 and S2).

### Study Procedures

At enrolment (day 0), a nasal swab for virological analysis was performed using a standard operating procedure (sample kit plus instructional video). Patients were allocated to treatment by a randomization list, with an arm ratio of 1∶1∶1, balanced by practitioner. A computer random number generator was used to select random permuted blocks of size 3. This randomisation code was given to the central hospital pharmacy that prepared blinded treatment units in conformity with good manufacturing practices (GMP). Each general practitioner received six treatment units and was told to distribute them by order of inclusion of his patients in the trial. Allocation was concealed through the similarity of all the containers and the impossibility for the GP to identify the treatment arm when opening the container. The three treatments were (1) oseltamivir capsule for oral use plus inhaled zanamivir, (2) oseltamivir plus inhaled placebo, (3) zanamivir plus oral placebo. Oseltamivir dosage was 75 mg orally twice daily; zanamivir dosage was 10 mg by oral inhalation using the commercialized GlaxoSmithKline Diskhaler, twice daily. Active drugs and placebo were kindly provided by Roche and Glaxo-SmithKline laboratories. A visiting nurse performed a nasal swab for virological analysis on day 2. Patients returned to their general practitioner at day 7 for a follow-up examination, and were contacted by phone on day 14. Patients, general practitioners assigning the patients, and outcome assessors (practitioners, virologists, patients), were blinded to treatment assignment throughout the study and statisticians until the end of the analysis.

### Virological Analysis

Nasal swabs placed into a transport medium (Virocult, Elitech) were transported at 4°C by special courier to the nearest National Influenza Centre (NIC) (Hospices Civils de Lyon, Lyon, or Pasteur Institute, Paris, France). Upon arrival, the swab samples were eluted into 2 ml of transport medium, processed for real-time reverse transcription (RT)-PCR analyses and inoculated onto MDCK cells for virus isolation and subsequent subtyping using a standard hemagglutination inhibition assay. For RT-PCR analyses, RNA extraction from 200 µl of specimen was performed using the QIAmp virus RNA mini kit (Qiagen) with RNA elution into a final volume of 60 µl. All real-time RT-PCR assays were performed in a final volume of 15 µL with 5 µL RNA, 0. µM of each primer, 0.2 µM probe, and 0.8 µl enzyme mix (SuperScriptIII platinum one-step quantitative RT-PCR system, Invitrogen). Type A influenza virus RNA was detected by a real-time RT-PCR targeting the conserved matrix gene using GRAM/7Fw (5′-CTTCTAACCGAGGTCGAAACGTA-3′) and GRAM/161Rv (5′-GGTGACAGGATTG GTCTTGTCTTTA-3′) primers and GRAM probe/52/+ (5′[Fam]-TCAGGCC CCTCAAAGCCGAG-[BHQ-1]3′) probe. The quality of the specimens was assessed by real-time RT-PCR targeting the GAPDH cellular gene [12]. Amplification was performed on a LightCycler 480 (Roche Diagnostics) (NIC, Pasteur Institute, Paris) or an ABI 7500 (Applied Biosystems) (NIC, Lyon). Cycling conditions are available upon request. Quantified synthetic RNA transcripts corresponding to the M and GAPDH genes were used as controls in parallel [13].

To take into account the variability in the quantity of cells collected by nasal swab, we calculated a normalized influenza viral load for each specimen; this normalized viral load was defined as the ratio of the M RT-PCR and GAPDH RT-PCR multiplied by the average GAPDH RT-PCR at day 0 to express results in copies of genome equivalent/µl (cgeq/µl). The virological response was defined as a normalized viral load below 200 cgeq/µl at day 2. This threshold was determined according to results on specimens from patients with a positive influenza A rapid test from winter 2008–2009 included in the French influenza surveillance network (GROG), and analysed by the 2NICs, and because it resulted in 5% false positive and 5% false negative results with respect to virus isolation (Table S1). It was validated by the independent data-monitoring committee prior to any analysis. Sensitivity of the qRT-PCR was assessed using serial dilutions of quantified synthetic transcripts corresponding to the target genes (Text S3). To assess comparability of the data between the two NICs, specimens were exchanged showing excellent concordance. The threshold of the qRT-PCR used was set well above the limit of detection.

### Clinical Response

Oral temperature was recorded and severity of seven symptoms (nasal stuffiness, sore throat, cough, muscle aches, tiredness or fatigue, headache, and feverishness) was rated by the patient twice daily (morning and evening) up to day 5 and then once daily on a four-point scale (0, none; 1, mild; 2, moderate; 3, severe) [2]–[4],[14]. The time to resolution of illness was defined as time from study drug initiation to time of symptom alleviation. Symptom alleviation was defined as the first 24-h period during which the above seven symptoms were absent or only mild as previously described [3],[4]. Influenza-related clinical events were defined as incidence of a secondary complication (such as pneumonitis or otitis) independently of any antibiotic initiation, and/or occurrence of exacerbation of a preexisting chronic disease. Patients reported treatment compliance using a self-administered questionnaire; full compliance during the day 0 to day 2 period was considered when 100% of planned drug intakes had been completed.

### Endpoints, Analyzed Populations

The primary efficacy endpoint was the proportion of patients with RT-PCR<200 cgeq/µl on day 2 of treatment. Given the viral shedding kinetics in patients with seasonal influenza receiving neuraminidase inhibitors, the day 2 virological endpoint was considered to be best suited to measurement of virological effects [2],[4]. Other endpoints were (1) the decrease of log_10_ viral load between days 0 and 2 in the patients with confirmed influenza A on day 0 and available samples both at days 0 and 2; (2) the time to resolution of illness; (3) the number of patients with alleviation of symptoms at the end of treatment (day 5); (4) the symptoms score at the end of treatment; (5) the incidence of secondary complications of influenza such as otitis, bronchitis, sinusitis, pneumonia, and the use of antibiotics; (6) the occurrence of adverse events in all participants having received at least one dose.

According to the protocol, the intention-to-treat (ITT) analysis was performed on two populations: (1) all enrolled patients (primary objective), and (2) enrolled patients with an influenza A virus infection confirmed by RT-PCR on day 0 (influenza A-infected population).

### Sample Size and Statistical Analysis

Sample size evaluation assumed that virological response was obtained in 70% of patients in the oseltamivir-zanamivir arm, compared to 55% in each of monotherapy arms on the basis of the extrapolation of the results of previous trials [2],[4]. Samples of 300 subjects per arm had 90% power to detect this difference, with a two-sided test and a type I error of 0.025 because of the two tests (factoring 20% lack of follow-up). Proportions of response at day 2 were compared between the combination therapy arm (OZ) and each monotherapy arm (O or Z) separately using two tests with a type I error of 0.025 because of the two tests. Patients without a day 2 sample were considered treatment failures. Mean decreases of log_10_ viral load were compared using a *t*-test in patients who had both day 0 and day 2 samples assuming a value of 0.5 cgeq/µl when RT-PCR was negative. For clinical endpoints nonparametric tests were used. Times to resolution of illness and symptoms score at the end of treatment were compared using Wilcoxon tests. If time to symptom alleviation was missing it was imputed to be 14 d, i.e., the end of the trial; 95% confidence intervals (95% CIs) of median differences were estimated by bootstrap. Probability of symptoms alleviation versus day of treatment was estimated using the Kaplan Meier method and was compared between groups with the log-rank test. Proportions of clinical events and of patients with alleviation of symptoms were compared using Fisher's exact tests. As an exploratory analysis, 95% CIs for differences of response between the two monotherapies were also estimated. All analyses were performed using SAS software, version 9.1 (SAS Institute).

### Early Study Termination

Recruitment was interrupted on March 15th 2009 at the end of the epidemic period in France after the enrolment of the first 541 patients and prior to the emergence of the 2009 H1N1 pandemic later in 2009. A second recruitment period was planned for the next winter (i.e., 2009–2010) to reach the number of 900 patients required. However, due to the emergence of the 2009 H1N1 pandemic virus, the independent data-monitoring committee, without any prior knowledge of the results, recommended terminating the trial and conducting an early analysis on May 6th 2009. This decision was based firstly on the need to rapidly provide results on efficacy and tolerance of combined oseltamivir-zanamivir therapy, and secondly, on the inadvisability of pooling the results of the two winters, one with a seasonal virus (winter 2008–2009, mainly H3N2) and the other one with a novel pandemic virus of a different subtype (winter 2009–2010).

## Results

### Patients

**Figure 1:**
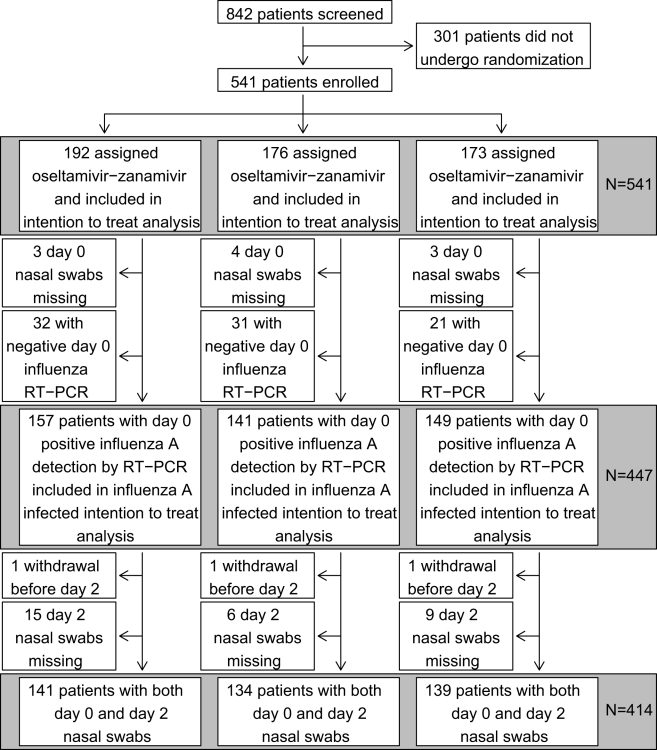
Trial flow chart.

Out of the 900 patients initially planned, a total of 541 patients were enrolled by 145 general practitioners. They were randomly assigned to oseltamivir plus zanamivir (OZ, *n* = 192), oseltamivir plus inhaled placebo (O, *n* = 176), zanamivir plus oral placebo (Z, *n* = 173) (Figure 1). Mean age was 39 y (standard deviation [SD] = 13), 49% were male, 14% had preexisting chronic diseases, mean fever was 38.2°C (SD = 0.8). The mean duration of illness before enrolment was 25 h (SD = 10). Other characteristics of patients appeared well balanced in the three arms (Table 1). The rate of fully compliant patients was not significantly different among the three arms (84% for the OZ arm, 88% for the O arm, and 85% for the Z arm).

**Table 1:** Characteristics of the 541 patients enrolled in the study and of the 447 influenza A-infected patients according to treatment arms.

Patients	Characteristics	Combined Oseltamivir and Zanamivir	Oseltamivir Plus Placebo	Zanamivir Plus Placebo
**All patients included in the study ** ***n*** ** = 541 (%)**		***n*** ** = 192 (35.5%)**	***n*** ** = 176 (32.5%)**	***n*** ** = 173 (32.0%)**
	Age (y): mean (SD)	38.7 (13.2)	39.5 (13.1)	39.9 (13.8)
	[Age range]	[18.3–73.2]	[18.1–76.3]	[18.0–84.2]
	*n* male (%)	91 (47.6%)	92 (52.3%)	86 (49.7%)
	*n* smoker (%)	34 (17.8%)	25 (14.2%)	26 (15.0%)
	*n* comorbidities (%)	27 (14.1%)	27 (15.3%)	23 (13.3%)
	*n* fever at enrolment≥38°C (%)	123 (69.9%)	118 (73.3%)	117 (75.5%)
	*n* initiation of treatment≤24 h after onset of symptoms (%)	92 (47.9%)	85 (48.3%)	101 (58.4%)
	Symptoms score per patient^a^			
	Mean (SD)	15.2 (2.8)	14.9 (3.2)	15.1 (3.2)
	% of maximal score: mean (SD)^b^	72.4% (13.4)	71.0% (15.2)	72.1% (15.4)
**Influenza A–infected patients ** ***n*** ** = 447 (%)**		***n*** ** = 157 (35.1%)**	***n*** ** = 141 (31.6%)**	***n*** ** = 149 (33.3%)**
	Age (y): mean (SD)	38.7 (13.2)	39.5 (13.0)	40.1 (14.1)
	[Age range]	[18.3–73.2]	[18.1–76.3]	[18.0–84.2]
	*n* male (%)	76 (48.7%)	73 (51.8%)	77 (51.7%)
	*n* smoker (%)	22 (14.1%)	15 (10.7%)	20 (13.4%)
	*n* comorbidities (%)	21 (13.4%)	20 (14.2%)	20 (13.4%)
	*n* fever≥38°C at enrolment (%)	101 (67.8%)	95 (70.9%)	104 (75.9%)
	*n* initiation of treatment≤24 h after onset of symptoms (%)	72 (45.9%)	68 (48.2%)	86 (57.7%)
	Symptoms score per patient^a^			
	Mean (SD)	15.6 (2.7)	15.3 (3.2)	15.5 (3.1)
	% of maximal score: mean (SD)^b^	74.2% (12.8)	72.7% (15.2)	73.8% (15.0)
	Influenza virus subtype			
	H1N1	9 (5.7%)	5 (3.5%)	7 (4.7%)
	H3N2	136 (86.6%)	130 (92.2%)	129 (86.6%)
	Not determined	12 (7.6%)	6 (4.3%)	13 (8.7%)

a Sum of the severity of the seven day 0 influenza symptoms (feverishness, nasal stuffiness, sore throat, cough, muscle aches, tiredness-fatigue, and headache) using a four-point scale [2],[14].

b The score is expressed as a percentage of the maximal score of 21.

### Virological Samples

Out of the 541 enrolled patients, 447 (83%) had a RT-PCR laboratory confirmation of influenza A virus infection on the day 0 specimen, with a mean viral load of 4.38 log_10_ cgeq/µl (interquartile range [IQR] 3.75–5.30). All the day 0 specimens were GAPDH RT-PCR positive with a mean value of 3.88 log_10_ copies/µl.

### Virological Endpoints

#### Primary endpoint

In the ITT analysis, considering the 541 enrolled patients with positive influenza A rapid test, the proportion of patients with a RT-PCR<200 cgeq/µl on day 2 of treatment was 52.6% in the oseltamivir-zanamivir arm, 62.5% in the oseltamivir monotherapy arm (*p* = 0.055, for the OZ versus O comparison, treatment effect comparison: −9.9%, [95% CI −19.9 to 0.2]), and 40.5% in the zanamivir monotherapy arm (*p* = 0.020, for the OZ versus Z comparison; treatment effect comparison: +12.1%, [95% CI 2.02–22.3]) (Table 2).

**Table 2:** Virological and clinical response according to treatment arms in the 541 enrolled patients, between day 0 and day 2 (ITT analysis).

Type of Response	Virological and Clinical Response Variables	Combined Oseltamivir and Zanamivir	Oseltamivir Plus Placebo	O+Z versus O		Zanamivir Plus Placebo	O+Z versus Z		O versus Z
				*p*-Value	Difference [95% CI]		*p*-Value	Difference [95% CI]	Difference [95% CI]^a^
	*n* **patients**	**192**	**176**			**173**			
**Virological**	**Primary virological endpoint**								
	Day 2 influenza RT-PCR<200 cgeq/µl (% patients)	52.6%	62.5%	0.055	−9.9% [−19.9 to 0.2]	40.5%	0.020	+12.1% [2.02–22.3]	+22.0% [12.1–32.0]
**Clinical**	Time to resolution of illness in days (median, IQR)	3.5 [2.5–14]	3.0 [2–7]	0.015	+0.5 [0.0–1.5]	4.0 [2.5–14]	0.78	−0.5 [−1.0 to 0.5]	−1.0 [−1.5 to −0.5]
	*n* (%) of patients with alleviation of symptoms at end of treatment	111 (57.8%)	122 (69.3%)	0.023	−11.5% [−21.3 to −1.7]	100 (57.8%)	1.00	+0.0% [−10.1 to 10.1]	+11.5% [1.7–21.3]
	Symptoms score at end of treatment (median, IQR)	3 [2–5]	2 [1–4]	0.0006	+1.0 [0.0–1.0]	3 [1–6]	0.79	+0.0 [−1.0 to 0.0]	−1.0 [−2.0 to −1.0]
	*n* (%) of patients with clinical event during treatment	26 (13.5%)	15 (8.5%)	0.14	+5.0% [−1.3 to 11.4]	23 (13.3%)	1.00	+0.3% [−6.7 to 7.2]	−4.8% [−11.2 to 1.6]
	Initiation of antibiotics	17 (8.9%)	10 (5.7%)		—	13 (7.5%)		—	—
	Pneumonia	2 (1.0%)	1 (0.6%)		—	0 (0.0%)		—	—
	Other	21 (10.9%)	14 (8.0%)		—	22 (12.7%)		—	—

a Exploratory analysis.

In the ITT analysis, considering the 447 influenza RT-PCR-confirmed patients, the proportions were 45.9% in the oseltamivir-zanamivir arm, 58.9% in the oseltamivir monotherapy arm (*p* = 0.025 for the OZ versus O comparison; treatment effect comparison: −13.0%, [95% CI −23.1 to −2.9]), and 33.6% in the zanamivir monotherapy arm (*p* = 0.028 for the OZ versus Z comparison; treatment effect comparison: +12.3%, [95% CI 2.39–22.2]) (Table 2). The same trends were observed in the 382 fully compliant influenza A-infected patients (Table S3), and in the 395 patients with H3N2 infection with proportions of 42.4% in the oseltamivir-zanamivir arm, 58.6% in the oseltamivir monotherapy arm, and 30.3% in the zanamivir monotherapy arm.

#### Other virological endpoints

In the 414 influenza RT-PCR confirmed patients with both day 0 and day 2 available specimens, the day 2 to day 0 decrease was 2.14 log_10_ cgeq/µl in the oseltamivir-zanamivir arm, 2.49 log_10_ cgeq/µl in the oseltamivir monotherapy arm, (*p* = 0.060 for the OZ versus O comparison; treatment effect comparison −0.35, [95% CI −0.8 to 0.07]), and 1.68 log_10_ cgeq/µl in the zanamivir monotherapy arm (*p* = 0.016 for the OZ versus Z comparison; treatment effect comparison: +0.46, [CI 95% 0.03–0.9]) (Table 2).

### Clinical Endpoints

**Figure 2:**
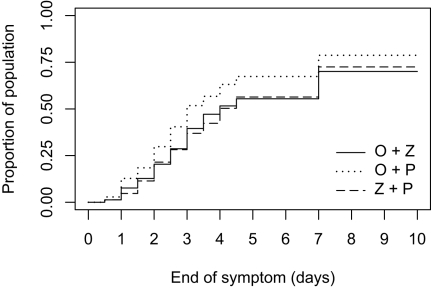
Proportion of the 447 influenza A-infected patients with alleviation of symptoms when treated with combined oseltamivir-zanamivir (plain line), oseltamivir plus placebo (dotted line), or zanamivir plus placebo (dashed line). Log-rank test for oseltamivir-zanamivir versus oseltamivir-placebo: *p* = 0.025 and for oseltamivir-zanamivir versus zanamivir-placebo: *p* = 0.036). Alleviation of symptoms defined by the presence of no symptoms of nasal stuffiness, sore throat, cough, muscle aches, tiredness-fatigue, feverishness, and headache or only mild ones, for at least 24 h.

The median time to resolution of illness in the 541 enrolled patients was 3.5 d in the oseltamivir-zanamivir arm, 3.0 d in the oseltamivir monotherapy arm (*p* = 0.015 for the OZ versus O comparison; treatment effect comparison: +0.5%, [95% CI 0.0–1.5]), and 4.0 d in the zanamivir monotherapy arm (*p* = 0.78 for the OZ versus Z comparison; treatment effect comparison: −0.5, [95% CI −1.0 to 0.5]) (Table 3). In the 447 influenza A-infected patients, this figure was 4.0 d in the oseltamivir-zanamivir arm, 3.0 d in the oseltamivir monotherapy arm (*p* = 0.018 for the OZ versus O comparison; treatment effect comparison: +1.0, [95% CI 0.0–4.0]), and 4.0 d in the zanamivir monotherapy arm (*p* = 0.96 for the OZ versus Z comparison; treatment effect comparison: +0.0, [95% CI −3.0 to 3.0]). Figure 2 presents the time to resolution of illness in the 447 patients, also showing by the log-rank test a significantly shorter time in the oseltamivir monotherapy arm. The time to resolution of illness was significantly shorter in patients with day 2 viral load below 200 cgeq/l (3.5 d) than in patients with viral load above 200 cgeq/l (7 d; *p* = 0.0014). The median symptoms score at day 5 (end of treatment) was 3 in the oseltamivir-zanamivir arm, 2 in the oseltamivir monotherapy arm (*p* = 0.013 for the OZ versus O comparison; treatment effect comparison: +1, [95% CI 0.0–1.0]), and 3 in the zanamivir monotherapy arm (*p* = 0.93 for the OZ versus Z comparison; treatment effect comparison: +0.0, [95% CI −1.0 to 0.0]) (Tables 2 and 3). Other clinical outcomes showed similar trends (Tables 2, 3, and S3).

**Table 3:** Virological and clinical response according to treatment arms in the 447 influenza A-infected patients between day 0 and day 2 (ITT analysis).

Type of Response	Virological and Clinical Response Variables	Combined Oseltamivir and Zanamivir	Oseltamivir Plus Placebo	O+Z versus O		Zanamivir Plus Placebo	O+Z versus Z		O versus Z
				*p*-Value	Difference [95% CI]		*p*-Value	Difference [95% CI]	Difference [95% CI]^a^
	*n* **patients**	**157**	**141**			**149**			
**Virological**	**Primary virological endpoint**								
	Day 2 influenza RT-PCR<200 cgeq/µl (%)	45.9%	58.9%	0.025	−13.0% [−23.1 to −2.9]	33.6%	0.028	+12.3% [2.39–22.2]	+25.3% [15.5–35.2]
	*n* **patients**	**141**	**134**			**139**			
	**Other virological endpoints in patients with available day 0 and day 2 nasal swabs (** ***n*** ** = 414)**								
	Mean (SD) viral load at day 0 (log _10_ cgeq/µl)	4.36 (1.36)	4.57 (1.32)			4.34 (1.37)			
	Mean (SD) viral load at day 2 (log _10_ cgeq/µl)	2.22 (1.12)	2.08 (1.17)			2.66 (1.35)			
	Mean (SD) viral load decrease between day 0 and 2 (log _10_ cgeq/µl)	2.14 (1.54)	2.49 (1.52)	0.060	−0.35 [−0.8 to 0.07]	1.68 (1.68)	0.016	+0.46 [0.03–0.9]	+0.81 [0.4–1.3]
	*n* **patients**	**157**	**141**			**149**			
**Clinical**	Time to resolution of illness in days (median, IQR)	4.0 [2.5–14]	3.0 [2–7]	0.018	+1.0 [0.0–4.0]	4.0 [2.5–14]	0.96	+0.0 [−3.0 to 3.0]	−1.0 [−4.0 to 0.0]
	*n* (%) of patients with alleviation of symptoms at end of treatment	87 (55.4%)	95 (67.4%)	0.043	−12.0% [−21.8 to −2.1]	84 (56.4%)	0.91	−1.0% [−11.1 to 9.2]	+11.0% [1.1 to 20.9]
	Symptoms score at end of treatment (median, IQR)	3 [2–5]	2 [1–4]	0.013	+1.0 [0.0–1.0]	3 [1–6]	0.93	+0.0 [−1.0 to 0.0]	−1.0 [−2.0 to −0.5]
	*n* (%) of patients with clinical event during treatment	19 (12.1%)	10 (7.1%)	0.17	+5.0% [−1.0 to 11.0]	18 (12.1%)	1.00	+0.02% [−6.6 to 6.7]	−5.0% [−11.0 to 1.0]
	Initiation of antibiotics	14 (8.9%)	7 (5.0%)	—	—	10 (6.7%)	—	—	—
	Pneumonia	2 (1.3%)	1 (0.7%)	—	—	0 (0.0%)	—	—	—
	Other	15 (9.6%)	9 (6.4%)	—	—	17 (11.4%)	—	—	—

a Exploratory analysis.

### Tolerance

Four serious adverse events occurred during the study, one of which was considered unrelated to study drugs (acute bacterial pneumonia at day 3 in a patient receiving oseltamivir-zanamivir combination). Two adverse events also occurred in patients receiving the oseltamivir-zanamivir combination: severe headaches leading to interruption of therapy and facial oedema following the first administration, disappearing within 24 h postdrug interruption. The remaining patient experienced repeated vomiting after oseltamivir monotherapy drug administration. All four patients completely recovered.

Other nonserious adverse events reported in more than 1% of the total population were in the OZ, O, and Z arms, respectively, nausea and/or vomiting (in 13, 4, and 5 patients), diarrhoea (in 2, 1, and 5 patients), and rash (in 1, 2, and 2 patients).

## Discussion

This large publicly funded clinical trial examined the effect of combination neuraminidase inhibitor antiviral therapy in influenza, as compared to each monotherapy plus placebo. It showed that, during the prepandemic winter of 2009 with a predominance of H3N2 viruses (more than 85%) in France, the oseltamivir-zanamivir combination seemed less effective than oseltamivir monotherapy, and not significantly more effective than zanamivir monotherapy in adults with seasonal influenza A virus infection.

Analysis of the different antiviral regimens' efficacy was based on a primary virological endpoint, which we hypothesized could be a sensitive and a more specific indicator than a primary clinical endpoint. Clinical endpoints, used as primary endpoints in previous studies, were used as secondary endpoints in the present study [1],[5]. Clinical endpoints, which are based on a global assessment of both general (mainly immunologically linked) and respiratory (mainly virologically linked) symptoms, are probably not the best way to monitor the virological effect of treatment, because clinical symptoms are not exclusive to influenza. We thus considered that a difference in viral shedding rate would be the best indicator of the virological effects of combined therapy, and consequently a valuable surrogate. Our initial hypothesis was that the combination of two antivirals may reduce the rate of resistant virus emergence (for a naturally susceptible pandemic virus and a nonimmune population). In addition, we hypothesized that for cases of infection with susceptible seasonal influenza viruses, this could not be easily shown, owing first to the rarity of this phenomenon in adults, and second, to the necessity of monitoring virus excretion for several days, whereas for cases of influenza due to H1N1 viruses, which are naturally resistant to oseltamivir, the question was not relevant. Given the viral shedding kinetics in patients with seasonal influenza receiving neuraminidase inhibitors, the day 2 virological endpoint was considered to be best suited to quantify virological effects. The 200 cgeq/l threshold was chosen, as it was the best compromise in terms of specificity and sensitivity as compared to standard culture. Of note, the same trends were observed when a 100 cgeq/l or a 1,000 cgeq/µl cut-off was used to define virological success (Table S2). Furthermore, the study was designed to be statistically two-sided to take into account the possibility that the combination would perform worse than either drug alone because of the theoretical concern of antagonism at the receptor level.

The oseltamivir-zanamivir combination seemed, both virologically and clinically, significantly less effective than the oseltamivir monotherapy. This result seems robust because (1) it was found using a double-blind placebo methodology, (2) there was overall concordance both among virological endpoints, and between virological and clinical endpoints, (3) it was confirmed over the three different subgroups of subjects included in the global population (541 enrolled patients, 447 influenza A-infected patients, 382 influenza A-infected and fully compliant patients). This lower clinical and virological response to the combination may suggest a negative effect of zanamivir on oseltamivir, as in the absence of interactions the effect of the combination should at least be additive [15]. A negative interaction at the level of binding at the catalytic pocket of the neuraminidase is an explanation that should be further investigated in vitro for both seasonal H3N2 and H1N1 viruses. Recent in vitro data showing the lack of synergy between oseltamivir and zanamivir, and some antagonism at higher concentrations of zanamivir on pandemic H1N1 2009 virus, are in agreement with this hypothesis [16]. Furthermore, contrary to oseltamivir, which upon digestive absorption needs to be metabolized, thus delaying arrival of the active drug at the infection site (*t*_max_ = 4 h), inhaled zanamivir is delivered directly to the primary site of influenza virus replication. The hypothesis that zanamivir is more likely to occupy the catalytic pocket first, thus preventing the action of oseltamivir, must be tested. According to this hypothesis, the combination would be largely reduced to a zanamivir monotherapy.

Whereas the results of the primary virological endpoints indicated a superiority of the oseltamivir-zanamivir combination to zanamivir monotherapy, clinical results were not significantly different, suggesting that oseltamivir adds essentially nothing to zanamivir monotherapy. This view is concordant with the above hypothesis of the predominant catalytic site occupation by zanamivir when the combination is administered.

As an exploratory analysis, oseltamivir showed a significantly higher clinical and virological efficacy as compared to zanamivir. This finding could be the consequence of a suboptimal treatment regimen in the zanamivir arm, since the IC50 values for the A(H3N2) viruses of the 2008–2009 season were 2- to 3-fold higher for zanamivir as compared to oseltamivir, but remained within the range for susceptible strains (GROG surveillance; NICs, unpublished data). The virological result is confirmed by the longer time to alleviation of the influenza symptoms in patients receiving zanamivir.

As the present study was conducted before the 2009 H1N1 pandemic during an influenza season where A(H3N2) viruses predominated, the impact of prepandemic seasonal A(H1N1) oseltamivir-resistant viruses on the results is expected to be negligible. We observed the same trends after excluding patients infected with seasonal H1N1 from our population. Of note, A(H3N2) viruses are to date sensitive to both drugs. It remains to be determined to what extent the present results can be extrapolated to susceptible viruses of other subtypes, e.g., H1N1, and in particular to the pandemic H1N1 2009 virus, which displays significant differences in the catalytic pocket of the neuraminidase [17], or to a mixed viral season with H3N2 and H1N1 cocirculating viruses.

We must acknowledge several limitations to our study. First, this preliminary analysis was conducted on a partial set of data after enrolment of 541 patients instead of the 900 initially planned. However, it is highly unlikely that the lower response of the combination as compared to oseltamivir would have been reversed if all originally planned 900 patients had been enrolled. Second, as previous randomized clinical trials had shown the superiority of each monotherapy as compared to placebo in terms of time to symptom alleviation and viral shedding, it was decided, on the basis of ethical reasons, that the study would not comprise a double placebo arm. Third, the proportion of patients with unavailable viral swab on day 2 was higher in the combination arm. As the missing value equals failure, this may have biased the results in the combination arm towards reduced performance. Indeed, in the analysis of the 414 patients with available day 0 and day 2 nasal swabs, the same trends were observed. Fourth, the virological response was assessed only in one site (nose) and at one time (day 2), which prevents extrapolation of the results to the entire virological response over time and throughout the respiratory tract. However, clinical endpoints completed the picture, giving information on the overall response. Fifth, as mentioned above, day 2 sampling was chosen to show the virological effect. However, this is probably not the best moment to look for resistance emergence induced by drug selective pressure, as it has been shown to occur later in the course of treatment [18]–[20]. Nevertheless, we looked for neuraminidase inhibitor resistance using a standard fluorimetric test in the 65 patients with day 2 positive viral culture; none of them carried a resistant virus, except for one patient infected with an H1N1 virus resistant to oseltamivir but susceptible to zanamivir, as were all H1N1 viruses circulating during the study period. However, the absence of resistance at day 2 does not rule out any further (post–day 2) resistance selection. Finally, this trial was conducted in adult outpatients, which prevents any extrapolation of the results to adults with severe presentation necessitating hospitalisation, and to children, who usually have more prolonged viral shedding. We chose the outpatient adult population because it seemed to be the most homogenous and the easiest in which to test our hypothesis.

Despite the theoretical potential for the reduction of the emergence of antiviral resistance, the lower efficiency of the oseltamivir-zanamivir combination found in this study calls for caution in its use in clinical practice. Thus, also considering the superiority of oseltamivir monotherapy over zanamivir monotherapy observed in this trial, oseltamivir should be the recommended primary anti-influenza treatment during influenza seasons with predominant H3N2 viruses naturally susceptible to oseltamivir. These results would need to be confirmed for the 2009 H1N1 pandemic virus and in the coming years, for future circulating influenza viruses.

## Supporting Information

Table S1 Proportion of false positive and false negative results for various viral load thresholds compared to viral isolation in the sample of GROG patients.
(0.04 MB DOC)

Table S2 Virological response in the 447 influenza A infected patients for thresholds of 100 and 1000 cgeq/µL.
(0.04 MB DOC)

Table S3 Virological and clinical response according to treatment arms in the 382 influenza A infected patients and fully compliant between day 0 and day 2 Intention to treat analysis.
(0.06 MB DOC)

Text S1 Methods. Choice of threshold for virological endpoint and sensitivity analysis.
(0.03 MB DOC)

Text S2 Trial protocol.
(0.59 MB PDF)

Text S3 CONSORT checklist.
(0.22 MB DOC)

## Abbreviations

cgeq: copies genome equivalent
CI: confidence interval
IQR: interquartile range
ITT: intention to treat
NIC: National Influenza Centre
O: oseltamivir
OZ: oseltamivir-zanamivir combination
RT: reverse transcription
SD: standard deviation
Z: zanamivir
