# Supplementary material for: Efficacy of Oseltamivir-Zanamivir Combination Compared to Each Monotherapy for Seasonal Influenza: A Randomized Placebo-Controlled Trial
Source: PLoS Med. 2010 Nov 2;7(11):e1000362. doi: 10.1371/journal.pmed.1000362 (PMC2970549; doi:10.1371/journal.pmed.1000362)
Supplement: Table S1 — Proportion of false positive and false negative results for various viral load thresholds compared to viral isolation in the sample of GROG patients. (0.04 MB DOC) [file pmed.1000362.s001.doc]

**Table S1:** Proportion of false positive and false negative results for various viral load thresholds compared to viral isolation in the sample of GROG patients

| **Threshold**  (cgeq/µL) | **Proportion of false positive**  (viral load > threshold and no viral isolation) | **Proportion of false negative**  (viral load < threshold and viral isolation) |
| --- | --- | --- |
| 0 | 6.5% | 0% |
| 2 | 6.5% | 0% |
| 4 | 6.5% | 0% |
| 10 | 6.5% | 1.6% |
| 20 | 6.5% | 1.6% |
| 40 | 6.5% | 1.6% |
| 100 | 4.8% | 1.6% |
| 200 | 4.8% | 1.6% |
| 400 | 4.8% | 3.2% |
| 1000 | 4.8% | 3.2% |

The threshold of 200 cgeq/µL was chosen by an independent data-monitoring committee from this set of results. For this threshold, 8 out of the 11 patients with negative virus isolation are below the threshold and only 1 out of the 51 patients with positive viral isolation is below the threshold. The independent data-monitoring committee also suggested performing sensitivity analyses in the BIVIR trial with thresholds of 100 and 1000 cgeq/µL. The comparison of the virological response in the 447 influenza A patients using these two other thresholds are given in Table S2. The same trend of lower response in the zanamivir group is observed for both thresholds (as observed in Table 2).
