# Supplementary material for: Efficacy of Oseltamivir-Zanamivir Combination Compared to Each Monotherapy for Seasonal Influenza: A Randomized Placebo-Controlled Trial
Source: PLoS Med. 2010 Nov 2;7(11):e1000362. doi: 10.1371/journal.pmed.1000362 (PMC2970549; doi:10.1371/journal.pmed.1000362)
Supplement: Table S2 — Virological response in the 447 influenza A infected patients for thresholds of 100 and 1000 cgeq/µL. (0.04 MB DOC) [file pmed.1000362.s002.doc]

**Table S2:** Virological response in the 447 influenza A infected patients for thresholds of 100 and 1000 cgeq/µL

|  | **Combined**  **oseltamivir and zanamivir** | **Oseltamivir**  **plus**  **placebo** | **O+Z**  **versus O**  P value  Difference between groups  [95% CI] | **Zanamivir**  **plus placebo** | **O+Z**  **versus Z**  P value  Difference between groups  [95% CI] | **O**  **Versus Z***  Difference between groups  [95% CI] |
| --- | --- | --- | --- | --- | --- | --- |
| **N**  **Number of patients** | **157** | **141** |  | **149** |  |  |
| Day 2 influenza RT-PCR < 100 cgeq/µL (%) | 40.1% | 45.4% | 0.36  -5.3%  [-15.4 ; 0.05] | 30.2% | 0.07  +9.9%  [0.2 ; 19.6] | +15.2%  [5.4 ; 25.0] |
| Day 2 influenza RT-PCR < 1000 cgeq/µL (%) | 65.6% | 72.3% | 0.21  -6.7%  [-16.2 ; 2.7] | 55.0% | 0.06  10.6%  [0.6 ; 20.5] | 17.3%  [7.6 ; 27.0] |
