# Supplementary material for: Efficacy of Oseltamivir-Zanamivir Combination Compared to Each Monotherapy for Seasonal Influenza: A Randomized Placebo-Controlled Trial
Source: PLoS Med. 2010 Nov 2;7(11):e1000362. doi: 10.1371/journal.pmed.1000362 (PMC2970549; doi:10.1371/journal.pmed.1000362)
Supplement: Table S3 — Virological and clinical response according to treatment arms in the 382 influenza A infected patients and fully compliant between day 0 and day 2 Intention to treat analysis. (0.06 MB DOC) [file pmed.1000362.s003.doc]

**Results Table S3**

Virological and clinical response according to treatment arms in the **382 influenza A infected patients** and **fully** compliant between day 0 and day 2 Intention to treat analysis.

|  | **Combined**  **oseltamivir and zanamivir** | **Oseltamivir**  **plus**  **placebo** | **O+Z**  **versus O**  P value  Difference between groups  [95% CI] | **Zanamivir**  **plus**  **placebo** | **O+Z**  **Versus Z**  P value  Difference between groups  [95% CI] | **O**  **Versus Z***  Difference between groups  [95% CI] |
| --- | --- | --- | --- | --- | --- | --- |
| **Virological response** |  |  |  |  |  |  |
| **Primary virological endpoint : influenza A infected patients and fully compliant from day 0 to day 2 (N=382)** | **131** | **124** |  | **127** |  |  |
| Day 2 influenza RT-PCR < 200 cgeq/µL (%) | 48.1% | 62.9% | 0.017  -14.8%  [-24.9 ; -4.8] | 37.0% | 0.079  11.1%  [1.0 ; 21.1] | 25.9%  [16.0 ; 35.8] |
| **Other virological endpoints : influenza A infected patients with available day 0 and day 2 nasal swabs influenza A infected patients and fully compliant from day 0 to day 2 (N=365)** | **123** | **121** |  | **121** |  |  |
| Mean (SD) viral load at day 0 (log _10_ cgeq/µL)  Mean (SD) viral load at day 2 (log _10_ cgeq/µL)  Mean (SD) viral load decrease between day 0 and 2 (log _10_ cgeq/µL) | 4.40 (1.28)  2.22 (1.15)  2.17 (1.51) | 4.58 (1.31)  2.02 (1.16)  2.56 (1.55) | 0.051  -0.4  [-0.8 ; 0.1] | 4.34 (1.37)  2.59 (1.37)  1.76 (1.71) | 0.044  +0.4  [0.0 ; 0.9] | +0.8  [0.3 ;1.3] |
| **Clinical response** |  |  |  |  |  |  |
| Time to resolution of illness in days (median, [IQR]) | 4.0 [2.5-14] | 3.0 [2-7] | 0.030  +1  [0.0 ; 1.5] | 4.0 [2.5-14] | 0.74  +0  [-1.0 ; 1.0] | -1  [-1.5 ; -0.25] |
| Number (%) of patients with alleviation of symptoms at end of treatment | 78 (59.5%) | 89 (71.8%) | 0.048  -12.2%  [-23.8 ; -0.7] | 76 (59.8%) | 1.00  -0.3%  [-12.3 ; 11.7] | +11.9%  [0.3 ; 23.6] |
| Symptoms score at end of treatment (median, IQR) | 3 [2-5] | 2 [1-4] | 0.008  +1  [0.0 ; 1.0] | 3 [1-5] | 0.85  0  [-1.0 ; 1.0] | -1  [-2.0 ; 0.0] |
| Number (%) of patients with clinical event during treatment   - antibiotics - pneumonia - other | 15 (11.5%)  11 (8.4%)  2 (1.5%)  12 (9.2%) | 9 (7.3%)  6 (4.8%)  1 (0.8%)  8 (6.5%) | 0.29  +4.2%  [-2.9 ; 11.3] | 14 (11.0%)  7 (5.5%)  0 (0.0%)  13 (10.2%) | 1.00  +0.4%  [-7.3 ; 8.1] | -3.8%  [-10.9 ; 3.3] |

**Note:**

O: oseltamivir, Z: zanamivir. CI: Confidence interval. SD : standard deviation

* Exploratory analysis
