# Supplementary material for: Efficacy of Oseltamivir-Zanamivir Combination Compared to Each Monotherapy for Seasonal Influenza: A Randomized Placebo-Controlled Trial
Source: PLoS Med. 2010 Nov 2;7(11):e1000362. doi: 10.1371/journal.pmed.1000362 (PMC2970549; doi:10.1371/journal.pmed.1000362)
Supplement: Text S1 — Methods. Choice of threshold for virological endpoint and sensitivity analysis. (0.03 MB DOC) [file pmed.1000362.s004.doc]

**Text S1**

**Methods: Choice of threshold for virological endpoint and sensitivity analysis**

As described in the methods section, for each specimen, the normalized influenza viral load was defined as the ratio of the M RT-PCR and GAPDH RT-PCR multiplied by the average GAPDH RT-PCR value at day 0 to express results in copies of genome equivalent/μL (cgeq/µL). The average of GAPDH RT-PCR values at day 0 was 25 565 copies/µL based on quantification using calibrated synthetic RNA transcripts (nt 6-231 of GAPDH gene). We defined the virological response as a normalized viral load below a threshold. As this was never done before, this threshold had to be set up. It has been selected using independent results of the 2 NICs on specimens collected by practitioners from patients analysed in the frame of the GROG (Groupes Régionaux d’Observation de la Grippe) network for routine surveillance in France. Briefly, nasal specimens collected from patients with a positive influenza A rapid test during the 2008-2009 season were used for implementation of this threshold. Overall, measurements of M RT-PCR and GAPDH RT-PCR from 62 patients were available, and we computed the normalised influenza viral load as in the BIVIR trial. We defined the threshold for viral load in cgeq/µL by comparison to results of virus isolation. Various thresholds were studied (from 0 to 1000 cgeq/µL) and the proportions of false positive and false negative detections compared to virus isolation were computed.

More precisely 11 out of 62 patients had negative viral isolation among which 4 had a non null viral load, i.e. 4/62 (6.5%) of false positive using a threshold of 0 cgeq/µL. Of note all patients with viral isolation had a positive viral load (i.e. 0/62=0% of false negative when the threshold is 0) in agreement with higher sensitivity of RT-PCR as compared to virus isolation. Table S1 reports the proportion of false positive and false negative for various thresholds.
