# Supplementary material for: Efficacy of Oseltamivir-Zanamivir Combination Compared to Each Monotherapy for Seasonal Influenza: A Randomized Placebo-Controlled Trial
Source: PLoS Med. 2010 Nov 2;7(11):e1000362. doi: 10.1371/journal.pmed.1000362 (PMC2970549; doi:10.1371/journal.pmed.1000362)
Supplement: Text S2 — Trial protocol. (0.59 MB PDF) [file pmed.1000362.s005.pdf]

ASSISTANCE PUBLIQUE HÔPITAUX DE PARIS

Essai AOM 06060 / Code Promoteur P060209

Essai randomisé en double insu comparant l'association oseltamivir et zanamivir à l'oseltamivir et placebo et au zanamivir et placebo dans le traitement curatif de la grippe de type A virologiquement suspectée en médecine ambulatoire

PROTOCOLE BIVIR

Promoteur Assistance Publique – Hôpitaux de Paris (AP- HP)
Direction de la Politique Médicale
Service de la Recherche Clinique et du Développement
carré historique de l'hôpital Saint-Louis
1, avenue Claude Vellefaux – 75010 paris
www.drrc.ap-hop-paris.fr

Investigateur Coordonnateur
Professeur Catherine LEPORT
Laboratoire de Recherche en pathologie infectieuse
Université Diderot- Paris 7
UFR de Médecine Site Bichat
16 Rue Henri Huchard
75018 PARIS

Service des maladies infectieuses
Groupe hospitalier Bichat-Claude Bernard
46, rue Henri Huchard - 75018 Paris
Tél. : 01 57 27 78 67 / 01 40 25 78 03
Fax : 01 57 27 76 91
Email : catherine.leport@bch.aphp.fr

Chef de projet DRCD
Aurélie GUIMFACK
DRCD - Hôpital Saint Louis
1, avenue Claude Vellefaux - 75010 Paris
Tél. : 01.44.84.17.98
Fax : 01.44.84.17.99.
Email : aurelie.guimfack@sls.aphp.fr

Méthodologiste
Pr France MENTRE
UMR 738 INSERM - Université Paris Diderot
Groupe hospitalier Bichat-Claude Bernard
46, rue Henri Huchard - 75018 Paris
Tél. : 01.57.27.77.59
Fax : 01.57.27.75.21
Email : France.mentre@inserm.fr

Version n°1.1 du 17 juillet 2008

LISTE DES PARTICIPANTS

Investigateur Coordonnateur

Pr. Catherine LEPORT –
Laboratoire de Recherche en pathologie infectieuse
Université Diderot- Paris 7
UFR de Médecine Site Bichat
16 Rue Henri Huchard
75018 PARIS

Service des maladies infectieuses
Groupe hospitalier Bichat-Claude Bernard
46, rue Henri Huchard - 75018 Paris
Tél. : 01 57 27 78 67 / 01 40 25 78 03
Fax : 01 57 27 76 91
Email : catherine.leport@bch.aphp.fr

Comité de pilotage

Laurent Andreoletti Laboratoire de Virologie, Hôpital R. Debré, EA-3798 Faculté de Médecine, Reims
Thierry Blanchon, Réseau Sentinelles, Inserm UPMC UMR S 707
Fabrice Carrat, Hôpital Saint-Antoine, APHP, Inserm UPMC UMR S 707
Xavier Duval, CIC Hôpital Bichat, APHP, Paris
Bruno Lina, Domaine Rockefeller, HCL, CNR du virus influenzae (Région Sud, Lyon)
Sandrine Loubière, Inserm unité 379, Institut Paoli-Calmettes, Marseille
France Mentré, INSERM et Université Paris 7 UMR738, UF de Biostatistiques, Hôpital Bichat, APHP
Anne Mosnier, Réseau des Grog, Paris
Annick Tibi, AGEPS, Pharmacie Centrale des Hôpitaux, Assistance Publique, Hôpitaux de Paris
Sylvie van der Werf, Institut Pasteur, CNR du virus influenzae (Région Nord, Paris)
Florence Tubach, URC Paris Nord, Groupe Hospitalier Bichat-Claude Bernard
Aurélie Guimfack, Délégation de la Recherche Clinique, AP-HP

Conseil Scientifique

Membres permanents

Antoine Flahault, Hôpital Tenon, APHP, Département de santé publique, Inserm UPMC UMR S 707
François Bricaire, APHP, Hôpital Pitié Salpétrière, Paris
Jean-Paul Moatti, Inserm unité 379, Institut Paoli-Calmettes, Marseille
Philippe Arnaud, Hôpital Bichat, APHP, Service de la Pharmacie
Jean-Marie Cohen, Réseau des Grog, Paris

Membres invités

Zeina Eid, laboratoires GSK
Christine Peurichard, laboratoires GSK
Mariem Charaf Eddine, laboratoires Roche
Michelle Pecking, laboratoires Roche
Anne Gysembergh-Houal, Délégation de la Recherche Clinique, AP-HP
Quatre représentants des médecins généralistes, dont un médecin Sentinelles et un médecin du Grog

Chef de projet de l'essai
Cécile Charlois Ou, LRPI, UFR de Médecine Site Bichat, 16 Rue Henri Huchard, Paris

Assistante chef de projet DRC
Christine Lanau, Délégation de la Recherche Clinique, AP-HP

Coordinateur d'essais cliniques URC
Caroline Quintin, URC Paris Nord, Groupe Hospitalier Bichat-Claude Bernard

Comité de vigilance

Geneviève Chêne, Inserm U897, Bordeaux 2
Claude Hannoun, virologie, Institut Pasteur
Daniel Vittecoq, service des maladies infectieuses et tropicales, hôpital Paul Brousse, AP-HP, Paris

Méthodologie et analyse statistique

Pr. France MENTRE
UMR738 - INSERM - Université Paris Diderot – Paris 7, UF de Biostatistiques
Hôpital Bichat - 46 rue Henri Huchard - 75018 Paris
Tél. : 01.57.27.77.59
Fax : 01.57.27.75.21
Email : France.mentre@inserm.fr

Représentants du Promoteur APHP

- **Département à la Recherche Clinique et au développement (DRCD)**

Hôpital Saint Louis
1, avenue Claude Vellefaux - 75010 Paris
Fax : 01.44.84.17.99.

Aurélie GUIMFACK
Tél. : 01.44.84.17.98
Email : aurelie.guimfack@sls.aphp.fr

Christine LANAU
Tél. : 01.44.84.17.89
Email : christine.lanau@sls.aphp.fr

- **Unité de Recherche Clinique Paris Nord**

Département d'Epidémiologie, de Biostatistique et de Recherche Clinique
Hôpital Bichat - 46 rue Henri Huchard - 75018 Paris
Fax : 01 40 25 67 73

Dr Florence TUBACH
Tél. : 01 40 25 79 41
Email : florence.tubach@bch.aphp.fr

Caroline QUINTIN
Tél. : 01 40 25 79 66
Email : caroline.quintin@bch.aphp.fr

Les produits délivrés dans cet essai (verum et placebo) sont fournis par les laboratoires Roche et GSK

Ce protocole contient des informations confidentielles rédigées par le comité scientifique de BIVIR et ne doit être utilisé que pour la conduite de l'essai. Le protocole ne doit pas être transmis à des personnes non concernées par l'essai, ni utilisé dans un autre but, sans l'accord écrit préalable des deux investigateurs principaux de l'essai.

CONFIDENTIEL

Page de SIGNATURE D'UN PROTOCOLE de recherche biomédicale
par l'investigateur COORDONNATEUR et le représentant du PROMOTEUR

Code de la Recherche biomédicale : ***P / AOM***

BIVIR : « Essai randomisé en double insu comparant l'association oseltamivir et zanamivir à l'oseltamivir et placebo et au zanamivir et placebo dans le traitement curatif de la grippe de type A virologiquement suspectée en médecine.»

Version N° 1.1 du 17 juillet 2008

L'investigateur coordonnateur :

Professeur Catherine LEPORT
Laboratoire de Recherche en pathologie infectieuse
Université Diderot- Paris 7
UFR de Médecine Site Bichat
16 Rue Henri Huchard
75018 PARIS

Service des maladies infectieuses
Groupe hospitalier Bichat-Claude Bernard
46, rue Henri Huchard - 75018 Paris

Date : 17……/…07……/…2008……..

Signature :

Le promoteur :

Docteur Olivier CHASSANY
Assistance publique – hôpitaux de Paris
Délégation Interrégionale à la Recherche Clinique
Hôpital Saint Louis
1, avenue Claude Vellefaux - 75010 PARIS

Date : ………/………/………..

Signature :

NB : cette version correspond au texte du protocole et annexes adressés au CPP et à l'autorité compétente respectivement pour avis et demande d'autorisation.

SOMMAIRE

1 RESUME SYNOPTIQUE DU PROTOCOLE 8

2 SCHEMA DE L'ETUDE 12

3 INTRODUCTION ET JUSTIFICATION DE L'ETUDE 13

4 OBJECTIFS DE L'ETUDE 22

4.1 Objectif principal 22

4.2 Objectifs secondaires 22

5 PLAN EXPERIMENTAL 23

5.1 Type de l'étude 23

5.2 Critères de jugement 23

5.3 Durée de l'étude 23

5.4 Médecins investigateurs et nombre prévu de sujets 24

5.5 Bras de l'essai 24

5.6 Critères d'éligibilité 24
5.6.1 Critères d'inclusion 24
5.6.2 Critères de non-inclusion 25

5.7 Evaluation pré-thérapeutique 25

5.8 Unité de traitement et kits de prélèvements 25

5.9 Visite d'entrée ou d'inclusion 26
5.9.1 Randomisation et désignation du numéro des sujets 26
5.9.2 Recueil des données 26
5.9.3 Dispensation des médicaments étudiés 26

5.10 Analyse des prélèvements naso-pharyngés 26

5.11 Administration des médicaments étudiés 27

5.12 Recueil des autoquestionnaires 27

5.13 Suivi des patients et recueil des données 27
5.13.1 Visite à J2 27
5.13.2 Visite à J7 27
5.13.3 Visite à J14 27

5.14 Schéma de la prise des traitements étudiés, des prélèvements et des consultations 28

5.15 Liste des données recueillies 30
5.15.1 Recueil à J0 par le médecin investigateur 30
5.15.2 Recueil J0 à J7 par le patient : autoquestionnaire remis au patient par son médecin généraliste investigateur et rendu à ce dernier à J7 30
5.15.3 Recueil à J7 par le médecin investigateur 30
5.15.4 Appel téléphonique à J14 par une infirmière de recherche clinique 31

6 ANALYSES STATISTIQUES ET ECONOMIQUES 32

6.1 Méthodes d’analyse statistique **32**
6.1.1 Comparabilité des groupes 32
6.1.2 Analyse du critère principal 32
6.1.3 Analyse des critères secondaires 32

6.2 Justification du nombre de sujets **33**

6.3 Extrapolation des conclusions **34**

7 EVALUATION DE LA SECURITE **35**

7.1 Description des paramètres d'évaluation de la sécurité **35**

7.2 Méthodes et calendrier prévus pour mesurer, recueillir et analyser les paramètres d'évaluation de la sécurité **35**
7.2.1 Comité de pilotage 35
7.2.2 Comité de vigilance 36

7.3 Procédures mises en place en vue de l'enregistrement et de la notification des évènements indésirables **36**
7.3.1 Evènements indésirables non graves 36
7.3.2 Evènements indésirables graves (EIG) 36

7.4 Modalités et durée du suivi des personnes suite à la survenue d'évènements indésirables **37**

8 DUREE DE L’ETUDE ET CALENDRIER **39**

9 DROIT D'ACCES AUX DONNEES ET DOCUMENTS SOURCE **40**

10 CONTROLE ET ASSURANCE DE LA QUALITE **41**

10.1 Procédures de monitoring **41**

10.2 Transcription des données dans le cahier d’observation **41**

11 CONSIDERATIONS LEGALES ET ETHIQUES **42**

11.1 Demande d’autorisation auprès de l’Afssaps **42**

11.2 Demande d’avis au Comité de Protection des Personnes **42**

11.3 Modifications **42**

11.4 Déclaration CNIL **43**

11.5 Note d’information et Consentement éclairé **43**

11.6 Rapport final de la recherche **43**

12 TRAITEMENT DES DONNEES ET CONSERVATION DES DOCUMENTS ET DES DONNEES RELATIVES A LA RECHERCHE **44**

13 ASSURANCE ET ENGAGEMENT SCIENTIFIQUE **45**

13.1 Assurance **45**

13.2 Engagement scientifique **45**

14 REGLES RELATIVES A LA PUBLICATION 46

15 ANNEXES 47

15.1 Engagements du promoteur et de l'investigateur 47

15.2 Cahier d'observation 47
15.2.1 Informations recueillies par le médecin généraliste ou l'infirmière investigateur 47
15.2.2 Informations recueillies par les virologues des deux CNR grippe (France Nord et France Sud) 48
15.2.3 Informations recueillies par auto-questionnaire (patient) 49

15.3 Lettre d'information au patient 50

15.4 Consentement de participation 56

16 REFERENCES 57

1 RESUME SYNOPTIQUE DU PROTOCOLE

Contexte

Actuellement, l'Organisation Mondiale pour la Santé (OMS) considère que nous sommes face à un risque potentiel majeur de pandémie grippale qui pourrait être dûe à l'émergence de nouveaux variants de virus Influenza A. Dans le contexte d'une prochaine pandémie à virus Influenza de type A, les antiviraux ont un rôle important à jouer lors de la première vague et permettraient de réduire de manière significative les taux de mortalité en traitant précocement les sujets présentant les premiers signes de l'infection. En France, le zanamivir, (laboratoires GSK) et l'oseltamivir (laboratoires Roche) disposent d'une indication dans le traitement curatif et prophylactique de l'infection grippale. Ces deux produits font l'objet de mesures de stockage par l'Etat français dans le cadre du plan de préparation contre une éventuelle pandémie grippale. Les essais princeps publiés concernant l'efficacité des monothérapies par oseltamivir ou zanamivir dans le traitement curatif de la grippe montrent que ces produits peuvent réduire la durée des symptômes de 24 heures avec une diminution significative des niveaux de charge virale dans les sécrétions nasopharyngées. Alors que la prévalence des virus résistants aux anti-neuraminidases était jusqu'à présent inférieure à 1%, au cours de l'épidémie de grippe saisonnière 2007-2008, ≈ 25% des souches A H1N1 circulant en Europe (≈ 50% en France) présentaient une résistance naturelle à l'oseltamivir, mais non au zanamivir, liée à la présence d'une mutation (H294Y) dans la neuraminidase. Cette résistance virologique n'a pas entraîné de résistance clinique au traitement des cas symptomatiques par oseltamivir (Lackenby A, 2008 ; Collins PJ, 2008). Afin de mieux définir les protocoles de prescription et d'utilisation des antineuraminidases dans le cadre d'une pandémie à Influenza A, et de réduire le risque d'émergence de souches résistantes, il parait opportun de déterminer s'il existe un bénéfice virologique (durée d'excrétion virale) et clinique (durée des symptômes) à l'utilisation d'une bithérapie par rapport à une monothérapie basée sur l'utilisation d'antineuraminidases. Même si les deux molécules appartiennent la même classe d'antiviraux, cette association pourrait avoir un effet synergique du fait de différences significatives entre les cinétiques de distribution et la biodisponibilité des deux produits. De plus, dans la perspective d'une pandémie avec un virus portant une neuraminidase de sous-type N1, il apparaît que les mutations conférant une résistance vis-à-vis des deux molécules ne sont pas strictement identiques. Dès lors, l'association de ces deux produits appartenant à la même classe peut avoir au moins trois intérêts : une réduction du portage et de l'excrétion du virus de la grippe dans les sécrétions respiratoires avec une diminution de la durée de contagiosité, une réduction significative de la durée des symptômes cliniques et de ses complications, et une réduction de la fréquence des mutations de résistances croisées ou non aux deux inhibiteurs de la neuraminidase. Afin d'évaluer l'impact virologique et clinique de l'association de deux inhibiteurs de la neuraminidase et de rationnaliser l'utilisation des ces antiviraux lors d'une pandémie, un essai randomisé en double insu, comparant l'association oseltamivir et zanamivir à l'utilisation de l'oseltamivir avec placebo et de zanamivir avec placebo dans le traitement curatif de la grippe A, sera réalisé en France métropolitaine.

Objectif principal

Comparer, en France métropolitaine chez des patients adultes symptomatiques et infectés par un virus influenza A, l'efficacité virologique de deux jours de traitement (J2) associant l'oseltamivir et le zanamivir par rapport à l'utilisation de l'oseltamivir avec placebo et du zanamivir avec placebo.

Méthodes

Plan expérimental

Essai randomisé en double insu testant l'efficacité virologique entre trois groupes de 300 patients. L'essai comparera en trois bras l'efficacité virologique de l'association oseltamivir et zanamivir à celle de l'oseltamivir avec placebo et de zanamivir avec placebo dans le traitement curatif de la grippe suspectée sur un test diagnostic rapide positif pour un virus Influenza de type A. L'essai sera conduit en période d'épidémie de grippe saisonnière, en médecine ambulatoire.

Bras 1: oseltamivir, 75 mg 2 fois/j, *per os* pendant 5 jours associé au zanamivir 5mg x 2 inhalations (soit 10mg) matin et soir, soit 20 mg par jour durant 5 jours.

Bras 2 : oseltamivir, 75 mg 2 fois/j, *per os* pendant 5 jours associé à un placebo correspondant à zanamivir 5mg x 2 inhalations (soit 10mg) matin et soir, soit 20 mg par jour durant 5 jours.

Bras 3 : zanamivir 5mg x 2 inhalations (soit 10mg) matin et soir, soit 20 mg par jour durant 5 jours associé à un placebo correspondant à oseltamivir (75 mg deux fois par jour pendant 5 jours)

Critères d'éligibilité

Critères d'inclusion

- Période de grippe saisonnière déclarée au niveau national déterminée par au moins l'un des deux réseaux de surveillance (réseaux Sentinelles ou GROG) participant à l'essai
- Patient présentant un syndrome grippal (cas suspect) comportant: fièvre ≥ 38°C (lors de la consultation, ressenti ou déclaré par le patient) avec au moins un symptôme respiratoire (toux, douleur pharyngée, rhinorrhée) et au moins un symptôme général (céphalée, asthénie, myalgies, frissons, ou sensation de malaise)
- Patient âgé de plus de 18 ans des deux sexes
- Patient paraissant capable de comprendre et remplir l'autoquestionnaire qui lui sera remis à l'issue de la consultation
- Patient dont la symptomatologie grippale a débuté depuis moins de 36 heures. Le début des symptômes est défini comme le premier constat ou ressenti fébrile
- Patient bénéficiaire de la sécurité sociale ou de la CMU
- Patient ayant eu un examen médical préalable
- Patient ayant un test diagnostique rapide positif pour une infection grippale de type A (cas probable)
- Patient volontaire pour participer à l'essai, ayant lu la lettre d'information et donné son accord écrit de participation à l'essai

Critères de non inclusion

- Patient vacciné contre la grippe pour la saison épidémique en cours
- Patient incapable d'utiliser correctement l'inhalateur de zanamivir
- Patient asthmatique ou présentant une broncho-pneumopathie obstructive en poussée, ou ayant une maladie chronique sévère en poussée

- Patient ayant pris des antiviraux (oseltamivir, zanamivir ou amantadine) dans les 14 jours qui précèdent l'inclusion
- Patient ayant des antécédents de dépression ayant nécessité la prescription d'antidépresseur, convulsion ou trouble psychiatrique grave
- Femme enceinte confirmée par un test de grossesse (bandelette urinaire) ou allaitante
- Patient présentant une insuffisance rénale connue (clairance de la créatinine ≤ 30 ml/min)
- Patient présentant une hypersensibilité connue à l'un des principes actifs ou excipients

Déroulement de l'essai

Visite de sélection : vérification de critères d'éligibilité, prélèvement nasal pour un test rapide de dépistage de la grippe A, pour les femmes test de grossesse par bandelette urinaire. Information et consentement du patient. Inclusion et randomisation du patient.

J0 : Recueil des informations cliniques, prélèvement nasal (écouvillon) pour recherche du virus Influenza, envoi au laboratoire de virologie référent, prise de la première dose de traitement et attribution des autres doses de traitement (durée de traitement : 5 jours), remise des autoquestionnaires.

J2 (après 4 prises de traitement, exceptionnellement 5 prises, si possible juste avant la prise suivante) : visite au domicile par une infirmière de recherche clinique, nouveau prélèvement nasal pour recherche du virus Influenza, envoi au laboratoire de virologie référent.

Les prélèvements virologiques seront adressés à l'un des deux CNR du virus Influenza (Régions Sud et Nord) pour recherche du virus grippal par RT-PCR temps réel standardisée, et culture de virus dès réception. Centralisation des prélèvements positifs en RT-PCR ou culture cellulaire Stockage des prélèvements congelés dans la perspective d'études virologiques complémentaires, notamment étude de l'apparition de sous-populations résistantes.

Autoquestionnaires remplis par le patient deux fois par jour jusqu'à J5, puis chaque jour jusqu'à J7 avec recueil des symptômes cliniques, de la tolérance, de l'observance de la prise des anti-viraux et des traitements associés.

J7 : visite de contrôle au cabinet du médecin investigateur, recueil des informations cliniques, des traitements pris, de la tolérance et récupération des autoquestionnaires.

J14 : contact téléphonique par une infirmière de recherche clinique, recueil de l'évolution clinique, des complications, de la prise d'antibiotiques, des arrêt de travail éventuels, du nombre de cas secondaires de grippe survenus dans le foyer du patient depuis l'inclusion et de la tolérance.

Critères de jugement

Le critère principal est l'efficacité virologique défini par une RT-PCR négative à J2 pour le virus Influenza de type A.

Les critères secondaires sont la durée et la sévérité des symptômes, la fréquence de survenue de syndromes grippaux à J14 chez les membres contact du foyer ne présentant pas de syndrome grippal antérieur ou concomitant à celui du patient inclus, les événements indésirables notifiés dans les trois bras de l'essai, l'observance thérapeutique, le nombre et la durée des arrêts de travail, la fréquence des complications de nature infectieuse (otite, bronchite, sinusite, et pneumonie), la consommation d'antibiotiques et le rapport coûts/efficacité du traitement. La fréquence des résistances aux antiviraux sera étudiée dans un second temps.

Méthodes statistiques

L'analyse principale sera réalisée en intention de traiter sur l'ensemble de la population des patients ayant une grippe de type A suspectée sur un test rapide positif, et ayant au moins reçu

une dose d'antiviral. Pour le critère principal, deux comparaisons seront effectuées : l'une entre le bras 1 (bithérapie) et le bras 2 (oseltamivir + placebo) et une entre le bras 1 (bithérapie) et le bras 3 (zanamivir + placebo), le risque de première espèce de chacun de ces tests sera de 0,025 afin de préserver une erreur globale de 0,05. Pour le critère principal, les proportions de patients ayant une RT-PCR négative à J2 entre les deux bras seront comparées par régression logistique ajustée sur le délai de prise de traitement après les premiers symptômes (moins ou plus de 24 heures). En cas de résultat virologique par RT-PCR manquant à J2, le prélèvement sera considéré comme positif (échec). Pour les critères secondaires, le risque de première espèce ne sera pas corrigé et sera de 0,05. Deux analyses complémentaires seront réalisées, l'une en intention de traiter sur la population des patients randomisés ayant une grippe clinique A virologiquement confirmée par RT-PCR à J0, l'autre per protocole, sur la population des patients randomisés ayant une grippe clinique A virologiquement confirmée par RT-PCR et ayant reçu l'ensemble du traitement prévu avant le prélèvement à J2.

Nombre de sujets nécessaires

Un effectif de 300 sujets par bras de randomisation, soit au total 900 sujets remplissant les critères d'inclusion donc ayant un résultat positif pour le test de détection rapide pour le virus Influenza de type A sera nécessaire pour avoir une puissance de 90% de mettre en évidence une différence de proportion de RT-PCR négative entre la bithérapie et chaque monothérapie avec un seuil de signification de 0,025 pour prendre en compte les deux tests bilatéraux. Ce calcul repose sur l'hypothèse que la proportion de RT-PCR négative à J2 est de 55% sous monothérapie et qu'elle est augmentée de 15 points, soit un total de 70%, sous bithérapie. Ce calcul conduit à 270 patients par bras, nombre qui a été augmenté de 10% pour prendre en compte l'ajustement sur le délai de traitement. Il faut noter que cet effectif aura aussi une puissance de plus de 90% de mettre en évidence une différence de 1 jour de durée des symptômes cliniques entre le bras bithérapie par rapport à chaque bras monothérapie (pour un écart-type de 3 jours). Dans la mesure où, sur un groupe de patients présentant une grippe clinique en période épidémique de grippe saisonnière, 60 à 70% ont un test de diagnostic rapide positif, il est prévu de proposer un test diagnostique rapide pour le virus Influenza de type A auprès d'environ 1450 patients éligibles pour que 900 sujets puissent participer à l'essai en cas de positivité.

Aspects réglementaires

Cette recherche sera soumise aux avis favorables préalables du CCP, de la CNIL et de l'AFSSAPS.

Durée de l'étude et calendrier

Les inclusions dans l'essai sont prévues après l'obtention des accords administratifs et financiers, au mieux durant la saison grippale 2008/2009. Il sera proposé aux médecins généralistes investigateurs de randomiser entre 6 et 12 patients. L'état des inclusions sera suivi en temps réel.

2 SCHEMA DE L'ETUDE

Contact et présentation de l'étude auprès des médecins libéraux investigateurs

↓

Ouverture des centres

↓

Information de l'investigateur de la période autorisée pour l'inclusion des patients

<u>Présélection</u> : Patient éligible et volontaire (1450 patients) (cas suspects)

Prélèvement respiratoire nasal pour dépistage d'une infection grippale de type A par un test de diagnostic rapide

↓

<u>Inclusion</u> : consentement écrit et randomisation (J0) (900 patients) des sujets avec un test diagnostic rapide positif pour le virus Influenza de type A (cas probable)

↓

Prélèvement respiratoire naso-pharyngé sur milieu de transport et envoi au laboratoire de virologie pour un diagnostic virologique par RT-PCR

↓

**Bras 1 : oseltamivir+zanamivir pendant 5 jours ;**	**Bras 2: oseltamivir+placebo pendant 5 jours ;**	**Bras 3: zanamivir +placebo pendant 5 jours ;**

↓

Visite au domicile par une infirmière de recherche clinique à J2, prélèvement respiratoire naso-pharyngé sur milieu de transport et envoi au laboratoire de virologie pour un diagnostic virologique par RT-PCR

↓

Autoquestionnaire de suivi de l'efficacité et la tolérance deux fois par jour jusqu'à J5 puis chaque jour jusqu'à J7

↓

Visite au cabinet du médecin investigateur à J7 et contact téléphonique à J14 par une infirmière de recherche clinique

↓

Saisie centralisée, analyse et présentation des résultats en comité scientifique

Restitution des résultats auprès des médecins libéraux et des patients.

Rapport final et publication scientifique des résultats de l'étude

3 INTRODUCTION ET JUSTIFICATION DE L'ETUDE

La grippe est une infection respiratoire aiguë, contagieuse, d'origine virale qui représente de nos jours l'une des principales causes de morbidité et de mortalité par maladie infectieuse en France. Maladie saisonnière, elle évolue le plus souvent sur un mode épidémique et peut toucher en France 5 à 15% de la population. L'incubation de la grippe a été estimée à 1,48 +/- 0,47 jour ; des données d'infection expérimentale ont montré que le début des symptômes coïncidait avec l'augmentation brutale de la réponse immunitaire ; l'excrétion virale étant à ce moment pratiquement à son pic. La période contagieuse est en moyenne de 2,6 jours (avec un intervalle de confiance à 95% de 2,1 à 3,0 jours) (Ferguson N, 2005). Les épidémies hivernales (grippe saisonnière) sont observées chaque hiver. Elles sont dues à de nouveaux variants évoluant constamment par glissement antigénique. Ces glissements ou variations antigéniques sont le reflet de modifications antigéniques mineures observées sur deux protéines de surface, l'hémagglutinine (H) et la neuraminidase (N) qui sont la cible principale de la réponse humorale. Ces modifications permettent au virus d'échapper très efficacement à la mémoire immunitaire. Chaque année, en France, entre novembre et mars, une épidémie de grippe d'ampleur nationale survient, conduisant 500 000 à 4 millions de personnes à consulter (Costagliola D, 1991 ; Carrat C, 1998), et provoquant entre 500 à 15 000 décès.

Exceptionnellement, un nouveau sous-type de virus influenza de type A peut être introduit chez l'homme. Ce phénomène a été observé trois fois au cours du 20e siècle. Cette introduction d'un nouveau virus peut résulter d'un réassortiment entre un virus grippal humain et un virus aviaire (ex : virus des pandémies de 1957 et de 1968), ou d'une adaptation d'un virus aviaire à l'homme par accumulation de mutations d'adaptation (ex : pandémie de 1918). Ces introductions de nouveaux sous-types (aussi appelées cassures antigéniques) sont responsables de pandémies dont l'impact a été considérable, tant en terme de mortalité que de morbidité. En effet, ces nouveaux sous-types émergent dans un terrain immunologiquement naïf au plan des anticorps, ce qui occasionne un taux d'attaque très élevé, des formes cliniques plus sévères et un taux de létalité plus important que lors de la grippe saisonnière.

La menace d'une pandémie grippale liée à l'apparition d'un nouveau virus de la grippe réside de plus dans la forte contagiosité (contagion par voie aérienne) et ce, avant même l'apparition de symptômes. Ce dernier point rend très difficile toute mesure de prévention, puisqu'il met en défaut la possibilité d'isoler les sujets contaminés dès qu'ils sont contagieux. Dans un contexte caractérisé par la grande mobilité de la population à l'échelle mondiale et l'importance des échanges notamment aériens entre les pays, les risques d'une dissémination mondiale rapide du virus avant qu'un vaccin adapté puisse être disponible sont d'emblée majeurs. Des systèmes d'alerte destinés à détecter précocement les flambées épidémiques sur la base de critères cliniques et d'indicateurs d'activité sanitaire, existent dans différents pays, comme en France, avec le concours de médecins généralistes des réseaux Sentinelles et GROG.

Depuis 2003, une épizootie de virus aviaire de sous-type H5N1 sévit en Asie puis en Europe et en Afrique, ayant fait à ce jour plus de 300 victimes humaines (http://www.who.int). Plusieurs centaines de millions de volailles d'élevage ont été sacrifiés ou vaccinés pour tenter d'enrayer l'épizootie, qui n'est toujours pas maîtrisée. Du fait de quelques rares cas de transmission à l'homme, l'OMS est en alerte ; cet état des lieux fait craindre une pandémie pouvant avoir des conséquences redoutables au niveau mondial.
Des modélisations mathématiques à partir des données épidémiologiques de la pandémie de 1918 ont été réalisées par l'Inserm et l'Institut de Veille Sanitaire. D'après ces estimations, entre

10 et 20 millions de Français pourraient être atteints lors de la première vague pandémique, ce qui en retenant un taux de létalité de 1 % pourrait conduire au décès de 100 à 200 000 individus.

Deux familles d'antigrippaux sont actuellement à la disposition du corps médical :
1/ Les inhibiteurs de la protéine M2 (amantadine et rimantadine) qui représentent le traitement historique de la seule grippe A. La fréquence élevée des effets indésirables et de la survenue de résistances rendent leur utilisation difficile et limitée en période épidémique. De plus, la majorité des virus aviaire A H5N1de clade 1, ainsi que certains virus H5N1 de clade 2, sont résistants à ces produits.
2/ Les inhibiteurs de la neuraminidase (INA) (oseltamivir et zanamivir) actifs sur les virus des grippe A et B. L'oseltamivir (Tamiflu®) et le zanamivir (Relenza®) ont obtenu en France l'autorisation de mise sur le marché (AMM) pour le traitement curatif de la grippe, indication limitée aux enfants à partir de 1 an pour l'oseltamivir et de 5 ans pour le zanamivir. L'oseltamivir est administré sous la forme de gélules à raison de 75 mg deux fois par jour pendant 5 jours chez l'adulte. Des gélules à 30 et 45 mg vont être disponibles pour le traitement des enfants de plus de 6 ans. Le zanamivir est administré par inhalation orale à l'aide du système Diskhaler® à la posologie de deux inhalations (2 x 5 mg) deux fois par jour, soit une dose totale de 20 mg par jour pendant 5 jours. L'oseltamivir et le zanamivir ont également une indication dans le traitement prophylactique de la grippe saisonnière. L'analyse des données de pharmacovigilance a mis en évidence pour l'oseltamivir des réactions anaphylactiques et anaphylactoïdes : dermatites, rash, eczéma, urticaires, réaction d'hypersensibilité et dans de très rare cas des réactions cutanées graves (syndromes de Steven-Johnson et érythème polymorphe), des nausées, vomissements, douleurs abdominales, élévation des transaminases, et plus récemment des convulsions et troubles neuro-psychiques (également décrits chez des patients grippés non traités). Pour le zanamivir, des rares cas de bronchospasme aigu et/ ou de diminution grave de la fonction respiratoire chez des patients ayant des antécédents de maladies respiratoires (asthme, bronchite chronique obstructive), et de très rares cas d'allergie, œdème facial, éruption cutanée, urticaire ont été décrits.

Les inhibiteurs de la neuraminidase sont actifs sur les souches de virus aviaire A H5N1 ayant sévi ou sévissant actuellement en Asie, Europe ou Afrique. L'oseltamivir est commercialisé en France par les laboratoires Roche. Le zanamivir est commercialisé par les laboratoires GSK. Ces deux produits font l'objet de mesures de stockage actuellement par l'Etat français, dans le cadre du plan de préparation contre une éventuelle pandémie grippale. Les essais princeps publiés concernant l'efficacité de ces produits, montre qu'elle est réelle mais modeste. La durée des symptômes est raccourcie en moyenne d'un jour chez l'adulte, passant de 5 jours sous placebo à 4 jours sous monothérapie. Au-delà de la réduction de la durée de la maladie, les antiviraux ont également un impact sur la réduction du portage viral nasal (MIST, 1998 ; Hayden FG, 1997 ; Nicholson KG, 2000 ; Treanor JJ, 2000).

Trois raisons nous incitent à évaluer l'efficacité et la tolérance d'une association d'antiviraux dans le traitement de la grippe A. La première est d'ordre épidémiologique, une meilleure efficacité démontrée d'une telle association, en particulier en termes de réduction de la durée de la période contagieuse, pourrait être de nature à reconsidérer la stratégie de l'usage des antiviraux afin de prévenir le plus efficacement possible la diffusion d'une souche pandémique. La seconde raison part de l'hypothèse plausible que, lors de l'utilisation intensive de ces produits, le taux de résistances à l'un des deux antiviraux (voire aux deux) devienne préoccupant, et de nature à menacer leur utilisation. Au cours de l'épidémie de grippe saisonnière 2007-2008 , ≈ 25% des souches A H1N1 circulant en Europe (≈ 50% en France) présentaient une résistance naturelle à l'oseltamivir, mais non au zanamivir, liée à la présence d'une mutation (H294Y) dans la neuraminidase. Cette résistance virologique n'a pas entraîné de

résistance clinique au traitement des cas symptomatiques par oseltamivir (Lackenby A, 2008, Collins JP, 2008). L'émergence de résistances pourrait être préoccupante, notamment au cours de la première vague pandémique lorsque le vaccin risque de ne pas être disponible. L'absence d'expérience de l'emploi d'une association de ces antiviraux, tant en terme d'efficacité que de tolérance, alors que l'on pourrait être conduit à envisager le recours à une telle association risque de conduire à des attitudes irrationnelles. La troisième raison relève du traitement de la grippe saisonnière, en particulier chez les personnes âgées et fragiles lorsque le vaccin n'a pas été administré ou se montre inefficace. Ces personnes, mais aussi toutes celles pour lesquelles ces deux produits sont actuellement indiqués en monothérapie par l'autorisation de mise sur le marché, pourraient bénéficier d'une association d'antiviraux si elle s'avérait plus efficace et aussi bien tolérée que leur utilisation en monothérapie. Il n'y a à notre connaissance aucun essai publié comparant l'association des deux produits dans le traitement curatif de la grippe de type A à l'heure de la rédaction de ce projet. Quoique leurs mécanismes d'action soient proches, il n'y a pas d'objection de principe à l'emploi de ces deux produits en association, dans le respect de leurs modalités d'administration. Ils s'administrent par deux voies différentes, l'une systémique orale, l'autre inhalée, endo-laryngée.

Nous pensons que l'utilisation d'un critère de jugement principal virologique, bien que les essais antérieurs démontrant l'efficacité des monothérapies aient eu pour critère de jugement la durée des symptômes, est justifiée par les arguments suivants :

- le critère clinique est un critère subjectif, dont la robustesse est probablement faible. Dans ces conditions le gain de 1 jour supplémentaire, par rapport au gain de 1 jour déjà obtenu avec les monothérapies comparativement au placebo parait incertain. Quant à un gain de plus faible ampleur sur la durée des symptômes, il n'est probablement pas cliniquement pertinent, et nécessiterait un nombre de sujets à inclure inaccessible.

- la corrélation entre la chronologie des symptômes et la dynamique virale dans les sécrétions respiratoires a été établie

- le mécanisme d'action de ces médicaments est une activité antivirale directe (action directe sur l'infection), l'effet sur les symptômes s'exerçant indirectement à travers cette activité biologique.

- l'effet attendu sur la réduction de la contagiosité passe directement par la réduction du portage et de l'excrétion du virus dans les sécrétions respiratoires

- le critère virologique apparaît comme un marqueur quantifiable, dont l'évolution précoce pourrait prédire la réponse clinique durant les premiers jours, et possiblement le risque de survenue de complications tardives

- le critère virologique apparaît novateur et ouvre des perspectives de quantification de virus dans les sécrétions respiratoires

Nous proposons de réaliser en France métropolitaine un essai randomisé contre placebo en double insu, comparant l'association oseltamivir et zanamivir à l'utilisation de l'oseltamivir avec placebo et de zanamivir avec placebo dans le traitement curatif de la grippe A.

Les essais randomisés publiés de monothérapies par anti-neuraminidases antigrippales

Nous présentons ci-dessous une analyse synoptique des cinq essais thérapeutiques contrôlés, publiés, et concernant les traitements curatifs par INA en monothérapie (oseltamivir et zanamivir). Les données que nous avons extraites de ces publications ont largement contribué à la justification des hypothèses, des définitions, et des critères de jugement retenus pour notre essai.

1. **Oseltamivir**

	**Treanor Jama 2000**		**Nicholson Lancet 2000**	
**Traitement**	Oseltamivir 75mg bid/150 mg bid/ placebo (5 jours)		Oseltamivir 75mg bid/150 mg bid/ placebo (5 jours) auto administration	
**Inclusion**	Non vacciné		Non Vacciné dans les 12 mois précédant, VIH, Maladies chroniques sévères en poussée, immunosuppresseurs, Toxicomanie, alcool	
	Age 18-65		Age 18-65	
	Fièvre ≥ 38°C		Fièvre ≥ 38°C	
	et ≥ 1 symptôme respiratoire		et ≥ 1 symptôme respiratoire (toux, douleur pharyngée, symptômes nasaux	
	et ≥ 1 symptôme général<br>et < 36 heures		et ≥ 1 symptôme général (céphalée, asthénie, myalgies, sueurs ou frissons, malaise)<br>et < 36 heures<br>Femme test gross neg et contraception	
**Nb de patients randomisés**	211/209/209		726 patients sans pathologie chronique sous-jacente<br>243/245/238	
**Sévérité**	7 symptômes :<br>toux, obstruction nasale, douleur pharyngée, asthénie, céphalée, myalgie, sensation fébrile<br>Echelle (0 à 3) : 0 absent, 3 sévère.<br>Recueil bid pdt 21 jours<br>Prise de température orale (thermomètre digital) bid et noté sur carnet<br>Autoévaluation pdt durée de traitement par échelle visuelle (11 graduations) de la capacité à réaliser activité de tous les jours<br>Echelle sur impression générale		Auto suivi<br>Température<br>Toux, obstruction nasale, douleur pharyngée, asthénie, céphalées, myalgies, sensation fébrile, (échelle de 0 absent, 1 minime, 2 modéré, 3 important) 2 fois par jour pendant 21 jours.<br>Pendant traitement (5 j), recueil chaque jours capacité à faire les activités normales, état général, qualité du sommeil (sur échelle de 10 points visuelle).	

	Treanor Jama 2000		Nicholson Lancet 2000	
	(prévalidé accessible en ligne) avant maladie et 1 fois par jours			
	Possibilité de prendre paracétamol (fourni), noté sur carnet		Paracétamol fourni, noté sur carnet	
	Observance : carnet et décompte des cp			
**% patients infectés**	59,6%		69%/66%/64% (culture et sérologie)<br>54%/56%/54% par culture seulement	
**Critère de Jugement**	- Durée résolution symptômes<br>- Sévérité maladie : score heures : aire sous la courbe (AUC)<br>- % bronchites, sinusites		- Durée résolution symptômes grippaux (à partir de J0 traitement)	
	Patients infectés (isolement virus ou séro conversion)<br>Soit 129/124/121 pts	Tous les patients	Patients infectés (isolement virus ou séro conversion J0-J21)	Tous les patients
**Durée des symptômes : placebo**	- 103,3 heures<br>- 963 score/heures	- 97 heures<br>- 887 score/heures	117 heures (traitement dans les 24h) ; 116.6 (Tnt dans les 36 heures).	116,1 heures
**Durée des symptômes : sous oseltamivir**	- 71,5 heures / 69,9 (réduction de 30%)<br>- 597 score/heures 626 score/heures<br>Réduction durée fièvre,<br>Retour à l'activité plus rapide (2 à 3 jours)	76,3 heures / 74,3 (réduction de 30%)<br>- 686 score/heures 629 score/heures	Diminution de 43h et 47h chez patients traités dans les 24h<br>74,5heures/ 70,7 heures<br>Patients traités dans les 3 heures :<br>Diminution de 29 heures pour 75mg (87,4 heures) et de 35h pour oseltamivir 150 mg (81,8h)	97,6/89,4 h
**Evénements indésirables**	Nausées vomissements :<br>7,4 % 3,4% (placebo) vs 18% 14% (oseltamivir)		Nausées : 12% (oseltamivir) vs 4% (placebo)	
**Virologie**	Prélèvements narines et pharynx J 0,3,5,7 (résultats chez 71, 67, 73 pts)<br>% de pts porteurs de virus et titre médian<br>- Placebo : 85% à J1, 28% à J3, 4% à J5, 0% à J7<br>- Oseltamivir 75 : 87% à J1, 28% à J3, 1% à J5, 0% à J7<br>- Oseltamivir 150 : 78% à J1, 27% à J3, 5% à J5, 0% à J7		Prélèvement narines et pharynx J 0, 2,4,6, 8<br>Calcul du titre viral (TCID 50/ML)<br>Réalisé chez 350 pts sur les 475 infectés.<br>Aire sous la courbe du titre viral médian plus faible de 30-40% pendant les 4 premiers jours 78,2 log 10 TCID 50xh/mL/ 94,4 vs 130,8	

	Treanor Jama 2000		Nicholson Lancet 2000	
	Titre médian : nul à partir de J3 dans les 3 bras<br>à J1 : 2,3 / 1,8/ 1,3			
**Durée des symptômes à inclusion**	32,6 /32,2/33,1 heures			
**Définition de la fin de la maladie**	Disparition des symptômes au début des 24 heures pendant lesquelles l'ensemble des symptômes sont cotés 1 ou moins et le restent pendant 24 heures		Disparition des symptômes au début des 24 heures pendant lesquelles l'ensemble des symptômes sont cotés 1 (minime) ou moins (absent) et le restent pendant 24 heures	
**Autres critères de jugement**	AUC : produit du score quotidien des symptômes et de la durée des symptômes comme définie ci-dessus. (scores-heures)		AUC : produit du score quotidien des symptômes et de la durée des symptômes<br>Besoin d'un traitement antibiotique pour complication<br>Portage viral	
**Analyse principale**	ITT au moins une dose reçue, chez patients infectés<br>Stratification région, tabac<br>Patients exclus censuré au moment du départ<br>Bonféroni pour critère principal<br>Kaplan Meyer sur disparition des symptômes<br>EI : analyse sur l'ensemble des patients		ITT au moins une dose reçue, chez patients infectés	
**Calcul de l'effectif**	Gain de 1,5 jours<br>SD de 3 jours<br>Puissance 80%, P=0.025		Echantillon de 190 pts pour avoir 85 infectés (50% de patients infectés), Puissance 80%, P=0.025<br>Formulation bilatérale avec seuil à 5% Distribution égale du niveau de significativité entre les comparaisons de chaque dose d'oseltamivir contre placebo, SD : 3 jours	

2. Zanamivir

ZANAMIVIR	Hayden NEJM 1997	The MIST Lancet 1998	Puhakka Scand J Infect Dis 2003
**Traitement**	Zanamivir inhalé vs Zanamivir intra nasal et inhalé vs placebo	Zanamivir 10 mg inhalé bid ou placebo (5 jours)<br>Auto administration	Zanamivir 10 mg inhalé bid ou placebo (5 jours)<br>Auto administration
**Inclusion**	Vaccinés exclus	Vacciné Non exclus	Vacciné Non exclus
	Age ≥ 12 ans	Age ≥ 12 ans	Age ≥ 18 ans (militaires)
	Fièvre ≥ 37.8°C et/ou sensation fébrile	Fièvre ≥ 37.8°C et/ou sensation fébrile	Fièvre ≥ 37.8°C et/ou sensation fébrile
	et ≥ 2 symptômes (toux, myalgies, douleur pharyngée, céphalée)	et ≥ 2 symptômes (toux, myalgies, douleur pharyngée, céphalée)	et ≥ 2 symptômes (toux, myalgies, douleur pharyngée, céphalée)
	et < 48 heures	et < 36 heures<br>Femme test grossesse négatif et contraception<br>Exclusion femmes allaitantes	et < 48 heures
**Nb de pts randomisés**	417 inclus	227/228	293/295
**Sévérité**	Auto-suivi	Auto suivi<br>Température (4 fois par jour) Toux, obstruction nasale, douleur pharyngée, asthénie, céphalées, myalgies, sensation fébrile, perte d'appétit (échelle de 0 absent, 1 minime, 2 modéré, 3 important) 4 fois par jour pendant 5 j de traitement ; puis 2 fois par jour pdt 9 jours ; qualité du sommeil. Quantité de prise de paracétamol<br>notée sur carnet	Auto-suivi
	Température (4 fois par jour) Toux, obstruction nasale, douleur pharyngée, asthénie, céphalées, myalgies, sensation fébrile, perte d'appétit (échelle de 0 absent, 1 minime, 2 modéré, 3 important)		
		Observance mesurée	
**% patients infectés**	63%	71%	74% (99% grippe A)
**Critère de Jugement**	- Durée résolution symptômes grippaux (entre J1 et J10)	- Durée résolution symptômes grippaux (à partir de J0 traitement)	- Durée résolution symptômes grippaux (à partir de J0 traitement)
	TDR+	Patients infectés (isolement virus ou séroconversion entre J0 et J21) ou TDR+	
**Durée des symptômes :**	5 jours	6,5 j	2,33 j

ZANAMIVIR	Hayden NEJM 1997	The MIST Lancet 1998	Puhakka Scand J Infect Dis 2003
**placebo**			3,83 j en l'absence de prise de paracetamol
**Durée des symptômes : sous zanamivir**	4 jours/4jours<br><br>Traitement plus efficace chez les patients traités dans les 30 heures (3 jours de symptômes en moins après début symptômes et chez les patients fébriles (2 jours de symptômes en moins).<br>Toux symptômes le plus persistant (4 jrs placebo, 3 jours zanamivir)<br>Durée de la fièvre courte mais prise d'antipyrétiques)	5 jours (gain 1,5 j)<br>Diminution de 2 jours chez les patients fébriles à l'inclusion<br>Retour à l'activité normale, durée anomalies du sommeil, utilisation d'antibiotiques plus courte ou moins fréquente dans bras zanamivir	2 j<br>3 j en l'absence de prise de paracetamol
**EI**	23% zanamivir inhalé et 25% zanamivir intra nasal et 18% placebo	Biologie à J0 et J5<br>37% sous zanamivir vs 43% sous placebo.<br>Bronchite (3% vs 7%), toux (4% vs 6%), sinusites (4% vs 1%), infections VRB (3% vs 3%) diarrhée (1% vs 4%)<br>Nausées : 2% vs 4% placebo	
**Viro**	Lavage nasal dans un sous groupe (J2, J4, J6, J8).<br>Titration virale (AUC).	Prélèvement narines (lavage et écouvillon, aspiration nasopharyngéeJ 0,<br>Test rapide	Prélèvement narine et gorge à J0 et 24h<br>Prélèvement gorge à 48h
**Durée des symptômes à inclusion**	31h	24,9 h	23,6 vs 24.5 h (pcb)
**Définition durée de la maladie**	Disparition des symptômes chez patient avec grippe prouvée.	Disparition des symptômes (fièvre < 37,8°C et céphalées minime, toux, myalgie et douleur pharyngée	Disparition des symptômes chez patient avec grippe prouvée.
**Autres critères de jugement**	Portage viral<br>4 jours dans bras traités versus 6 jours chez placebo<br>Diminution du titre infectieux de 2,1 log 10 TCID 50 /mL à D2 et 1,5 à D4 (significatif)<br>Pas de diminution dans les prélèvements de narines chez ceux recevant le Zanamivir inhalé)	PAS dAUC<br>Score sur une échelle de 100 points (pas détaillée)	% de patients à prélèvements virologiques négatifs<br>- à 24H (PCR négative seuil 3.34 log 10 copies/ml) : 72/212 (34%) zana vs (54/202) pcb (p=0.034)<br>- à 48H<br>118/212 (56%) zana vs 59/202 (29%) pcb (p<0.001)

ZANAMIVIR	Hayden NEJM 1997	The MIST Lancet 1998	Puhakka Scand J Infect Dis 2003
**Analyse principale**	Durée des symptômes	Suivi 28 jours<br>Définition de patients à haut risque (comorbidités, asthmatiques, pathologie cardiovasculaire autres qu'hypertension) sont inclus, sous analyse. Durée des symptômes plus longue 8 j placebo versus 5,5 j sous zanamivir (non significatif car petit effectifs (39 et 37 patients). Moins de complications sous zanamivir	Durée des symptômes
**Calcul de l'effectif**	Symptômes majeurs disparus à J6 chez 50% des patients infectés sous placebo.<br>Différence cliniquement parlante de 75% des patients avec absence de symptômes à J6<br>Test bilatéral 5%, Puissance 80%<br>Taux d'infection de 70%<br>Analyse de 2 études une Europe, une USA car problèmes d'inclusion.	Gain cliniquement significatif de 1 jour de durée de symptômes. Puissance 90%, pour test bilatéral avec 5% de seuil de sinification<br>SD : 2,75 jours<br>Effectif non calculé sur patients infectés.	Effectif non calculé sur patients infectés.

3. Comparaison oseltamivir - zanamivir

	Sugaya N (***Options for the Control of Influenza VI* conference Toronto, June 17-23, 2007**) Abstract O 65		
	**oseltamivir**<br>Enfants **n= 47**		**zanamivir**<br>Enfants **n = 25**
Age moyen:	7 ans		8,1 ans
Doses	adaptées aux poids		5 mg x 2 matin et soir
Durée	5 j		5 j
Durée fièvre sous traitement :	1.5 +/- 0.9 j		1.5 +/- 0.7 j
Présence virus gorge (H3N2)			
Au 3ème jour de Traitement :	80%		54 %
Au 5ème jour de Traitement :	47 %		8 %

Au total, par analogie avec ce qui a été observé dans d'autres infections, l'association de 2 antiviraux dans le traitement curatif de la grippe pourrait avoir plusieurs avantages : réduire la durée des symptômes et la période de contagiosité ; limiter la sélection de souches virales résistantes ; conserver une activité antivirale sur des souches virales de moindre sensibilité à l'un des 2 antiviraux ; accélérer la vitesse d'élimination du virus dans l'organisme humain.

4 OBJECTIFS DE L'ETUDE

4.1 Objectif principal

Comparer, en France métropolitaine chez des patients adultes symptomatiques et infectés par un virus influenza A, l'efficacité virologique de deux jours de traitement (J2) associant l'oseltamivir et le zanamivir par rapport à l'utilisation de l'oseltamivir avec placebo et du zanamivir avec placebo.

4.2 Objectifs secondaires

Cliniques :

Evaluer et comparer entre J0 et J2 chez les patients adultes inclus dans chacun des bras de l'étude (oseltamivir et zanamivir vs oseltamivir avec placebo vs zanamivir avec placebo) :

- la réduction de la durée et la sévérité des symptômes grippaux et leurs complications cliniques
- le taux d'attaque secondaire par mesure du nombre de cas de syndromes grippaux survenant chez les contacts du foyer du sujet inclus dans les 7 jours de la consultation,
- le nombre et la nature des événements indésirables notifiés dans les deux bras de l'essai,
- l'observance thérapeutique,
- le nombre et de la durée des arrêts de travail attribuables à la grippe,
- la fréquence de complications de nature infectieuse (otite, sinusite, bronchite, pneumonie),
- la consommation d'antibiotiques,
- le rapport coûts/efficacité.

Virologiques :

Détecter et préciser la prévalence des mutations de résistance aux inhibiteurs de la neuraminidase (oseltamivir et zanamivir) et leur distribution temporo-spatiale au cours de la saison épidémique 2008-2009 dans les différentes régions Françaises ayant inclus des patients.

Comparer phénotypiquement les niveaux de résistance des souches virales portant au moins une ou des mutations de résistance aux inhibiteurs de la neuraminidase avant (J0) et après deux jours de traitement (J2) associant l'oseltamivir et le zanamivir par rapport à l'utilisation de l'oseltamivir avec placebo et du zanamivir avec placebo

Analyser phylogénétiquement la distribution temporo-spatiale des souches après amplification et séquençage complet des gènes HA et NA des souches virales isolées au cours de l'étude à J0 et à J2

Evaluer les dérives génétiques et antigéniques (gènes HA et NA) des souches virales de virus Influenza A au cours de l'évolution de l'épidémie (Taubenberger et al., Nature 2005).

Etudier la diversité génétique par une analyse moléculaire des quasi-espèces virales en étudiant les séquences nucléotidique du segment HA1 du gène HA pour une sélection de souches et ceci avant (J0) et après traitement (J2) par les antineuraminidases (De Jong et al., 2007).

5 PLAN EXPERIMENTAL

5.1 Type de l'étude

Il s'agit d'un essai randomisé évaluant en double insu une bithérapie contre deux monothérapies plus placebo en trois groupes parallèles.

5.2 Critères de jugement

Le critère principal est l'efficacité virologique définie par une RT-PCR négative à J2 pour le virus Influenza de type A.

Les critères secondaires sont :

- la durée et la sévérité des symptômes,
- la fréquence de survenue de syndromes grippaux à J14 chez les membres contact du foyer ne présentant pas de syndrome grippal antérieur ou concomitant à celui du patient. L'analyse sera faite en considérant uniquement les cas secondaires, i.e. survenue dans les 7 jours de la consultation du cas index d'un syndrome grippal chez un sujet n'ayant pas *a priori* présenté de grippe depuis le début de la saison épidémique au jour de la consultation du sujet index et en intégrant l'ensemble des cas du foyer,
- les événements indésirables notifiés dans les trois bras de l'essai,
- l'observance thérapeutique,
- le nombre et la durée des arrêts de travail,
- la fréquence des complications de nature infectieuse (otite, bronchite, sinusite, et pneumonie),
- la consommation d'antibiotiques,
- le rapport coûts/efficacité du traitement,
- la prévalence des mutations de résistance aux inhibiteurs de la neuraminidase (oseltamivir et zanamivir),
- les niveaux de résistance des souches virales portant au moins une ou des mutations de résistance aux inhibiteurs de la neuraminidase avant (J0) et après deux jours de traitement (J2),
- les taux de dérives génétiques et antigéniques (gènes HA et NA) des souches virales de virus Influenza A au cours de la saison épidémique,
- la diversité génétique par une analyse moléculaire des quasi-espèces virales en étudiant les séquences nucléotidiques du segment HA1 du gène HA pour une sélection de souches et ceci avant (J0) et après traitement (J2) par les antineuraminidases.

5.3 Durée de l'étude

Pour chaque sujet inclus, la durée du traitement sera de 5 jours (soit 10 prises d'antiviraux). Le sujet sera suivi par le médecin investigateur qui le verra à l'inclusion J0 (jour de l'inclusion) et à J7. Le sujet effectuera un auto-suivi de ses symptômes sur une durée de 7 jours. Il rapportera son observance thérapeutique durant les 5 jours de traitement. Il sera suivi par une infirmière à J2 et J14 +/-2. La durée maximale de participation à l'étude d'un sujet sera de 16 jours.

5.4 Médecins investigateurs et nombre prévu de sujets

Plusieurs réseaux de surveillance en médecine générale ont été sollicités, parmi eux le réseau des Groupes Régionaux d'Observations de la Grippe (GROG) et le réseau Sentinelles de l'Inserm. Ces réseaux sont chargés d'identifier en France métropolitaine 300 médecins généralistes libéraux volontaires pour participer à l'essai. Ces médecins assureront à J0 le recrutement, l'inclusion des patients, le prélèvement et la distribution des traitements, et le suivi à J7.

Dans la mesure où, durant la période épidémique de la grippe, les patients présentant des signes évocateurs d'une infection grippale testés avec un test diagnostic rapide présentent un taux de positivité de l'ordre de 60% à 70%, il est prévu de proposer un test diagnostique rapide pour le virus Influenza de type A auprès d'environ 1450 patients éligibles pour participer à l'essai en cas de positivité. Il devrait ainsi être possible de randomiser 900 patients ayant un test diagnostique positif pour la grippe A, soit 300 patients dans chaque bras de l'essai. Chaque médecin investigateur pourrait être amené à présélectionner entre 9 et 18 patients, et randomiser entre 6 et 12 patients. La justification statistique de l'effectif nécessaire est donnée au § 6.2.

5.5 Bras de l'essai

Bras 1: oseltamivir, 75 mg 2 fois/j, *per os* pendant 5 jours associé au zanamivir 5mg x 2 inhalations (soit 10mg) matin et soir, soit 20 mg par jour durant 5 jours.

Bras 2 : oseltamivir, 75 mg 2 fois/j, *per os* pendant 5 jours associé à un placebo correspondant à zanamivir 5mg x 2 inhalations (soit 10mg) matin et soir, soit 20 mg par jour durant 5 jours.

Bras 3 : zanamivir 5mg x 2 inhalations (soit 10mg) matin et soir, soit 20 mg par jour durant 5 jours associé à un placebo correspondant à oseltamivir (75 mg deux fois par jour pendant 5 jours)

5.6 Critères d'éligibilité

5.6.1 Critères d'inclusion

- Période de grippe saisonnière déclarée au niveau national déterminée par au moins l'un des deux réseaux de surveillance (réseaux Sentinelles ou GROG) participant à l'essai
- Patient présentant un syndrome grippal (cas suspect) comportant: fièvre ≥ 38°C (lors de la consultation, ressenti ou déclaré par le patient) avec au moins un symptôme respiratoire (toux, douleur pharyngée, rhinorrhée) et au moins un symptôme général (céphalée, asthénie, myalgies, frissons, ou sensation de malaise)
- Patient âgé de plus de 18 ans des deux sexes
- Patient paraissant capable de comprendre et remplir l'autoquestionnaire qui lui sera remis à l'issue de la consultation
- Patient dont la symptomatologie grippale a débuté depuis moins de 36 heures. Le début des symptômes est défini comme le premier constat ou ressenti fébrile
- Patient bénéficiaire de la sécurité sociale ou de la CMU
- Patient ayant eu un examen médical préalable

- Patient ayant un test diagnostique rapide positif pour une infection grippale de type A (cas probable)

- Patient volontaire pour participer à l'essai, ayant lu la lettre d'information et donné son accord écrit de participation à l'essai

5.6.2 Critères de non-inclusion

- Patient vacciné contre la grippe pour la saison épidémique en cours

- Patient incapable d'utiliser correctement l'inhalateur de zanamivir

- Patient asthmatique ou présentant une broncho-pneumopathie obstructive en poussée, ou ayant une maladie chronique sévère en poussée

- Patient ayant pris des antiviraux (oseltamivir, zanamivir ou amantadine) dans les 14 jours qui précèdent l'inclusion

- Patient ayant des antécédents de dépression ayant nécessité la prescription d'antidépresseur, convulsion ou trouble psychiatrique grave

- Femme enceinte confirmée par un test de grossesse (bandelette urinaire) ou allaitante

- Patient présentant une insuffisance rénale connue (clairance de la créatinine ≤ 30 ml/min)

- Patient présentant une hypersensibilité connue à l'un des principes actifs ou excipients

5.7 Evaluation pré-thérapeutique

Le médecin investigateur, lorsque le patient rassemblera l'ensemble des critères d'éligibilité, pratiquera un prélèvement nasal pour un test diagnostique rapide du virus Influenza de type A.

Le formulaire de consentement sera remis au patient, qui disposera du temps de l'attente du résultat du test rapide (10 à 15 minutes) pour lire la note d'information et signer son consentement en cas de test positif pour la grippe A (cas probable). Les médecins généralistes investigateurs procèderont au recueil du consentement éclairé écrit des patients éligibles.

Pour les femmes, une fois le consentement signé un test de grossesse par bandelette urinaire sera effectué.

Ne seront randomisés que les patients volontaires remplissant l'ensemble des critères d'éligibilité pour l'inclusion (cas suspects), ayant un test diagnostic rapide positif vis-à-vis du virus Influenza de type A (cas probable) et, pour les femmes, ayant un test de grossesse négatif.

Les patients qui ne rempliront pas l'ensemble critère d'éligibilité ne seront pas inclus dans l'essai et recevront les soins habituels prodigués par leur médecin.

5.8 Unité de traitement et kits de prélèvements

Dans chacun des cabinets médicaux, des Unités de Traitement (UT) seront mises en stock au démarrage du protocole. Ces UT seront constituées du regroupement sous film rétractable de 2 boîtes portant le même numéro de traitement :

- une boîte de 10 gélules d' oseltamivir 75 mg ou de placebo d'aspect identique
- une boîte contenant 5 Rotadisks (de 4 doses de zanamivir 5mg ou de placebo) et 1 Diskhaler®.

Les kits de diagnostic rapide et de prélèvements nécessaires à l'essai seront simultanément mis à la disposition de chacun des investigateurs.

Les réapprovisionnements nécessaires seront réalisés en fonction des inclusions réelles de chaque investigateur.

Les médecins généralistes investigateurs attribuent aux patients les médicaments de l'essai alloués par le tirage au sort (numéro de traitement affecté à chaque patient lors de la randomisation); médecins et patients restent à l'insu du traitement attribué jusqu'à la fin de l'essai.

5.9 Visite d'entrée ou d'inclusion

5.9.1 Randomisation et désignation du numéro des sujets

Après vérification de l'ensemble des critères d'éligibilité, obtention du résultat positif du test rapide, recueil du consentement éclairé écrit du patient et pour les femmes obtention du résultat négatif du test de grossesse , le médecin investigateur procèdera à la randomisation du patient. Un numéro de sujet et de traitement sera alors attribué au patient.

En fonction du groupe de randomisation, le médecin en insu, comme son patient, attribuera un lot de traitement, dont le numéro lui a été communiqué lors de la randomisation.

Un prélèvement naso-pharyngé sera réalisé à l'aide d'un kit de prélèvement virologique spécifique, commercialisé et prêt à l'emploi. Une fois réalisé, le prélèvement sera conditionné et envoyé au laboratoire de virologie selon les procédures déjà établies par les CNR de la grippe.

5.9.2 Recueil des données

Le médecin remplira dans le cahier d'observation le formulaire de la visite d'inclusion, dont la première page permettra de confirmer la validation de l'ensemble des critères d'éligibilité.

Le médecin remettra les autoquestionnaires à son patient en lui expliquant les modalités de remplissage.

Il déterminera les rendez-vous prévus à J2 (infirmière à domicile), à J7 (MG au cabinet) et à J14 (contact téléphonique par l'infirmière).

5.9.3 Dispensation des médicaments étudiés

La première prise du traitement de l'essai sera administrée au patient par le médecin investigateur, qui lui fournira les explications nécessaires (posologie, utilisation du Diskhaler®, rythme des prises) et saisira l'heure de la première prise dans le cahier d'observation.

Le médecin investigateur remettra les autres doses de traitement de l'essai à son patient en lui expliquant les modalités d'auto-administration, pour une durée de 5 jours.

5.10 Analyse des prélèvements naso-pharyngés

Au niveau des deux laboratoires de virologie des CNR de la grippe, le diagnostic virologique sera confirmé par une « RT-PCR en temps réel standardisée » ciblant le gène M des virus grippaux de type A. Des contrôles de qualité des analyses de RT-PCR en temps réel seront réalisés préalablement à la mise en place de l'étude. Au cours de l'étude des contrôles de qualité internes seront réalisés et ceci dans les deux laboratoires CNR (Nord et Sud) de la grippe.

L’analyse par RT-PCR temps réel réalisée a J0 et J2 sur le prélèvement nasal permettra une évaluation quantitative du titre viral en nombre de copies de génome viral normalisé par rapport à la GAPDH .

Un isolement par des procédures classiques de culture cellulaire sera réalisé selon les techniques en vigueur dans les CNR et ceci afin d’isoler les souches de grippe A. Les souches virales isolées seront identifiées par typage/sous-typage/caractérisation du variant viral par test classique d’inhibition de l’hemagglutination ainsi que le cas échéant par séquençage moléculaire des gènes HA et NA.

Secondairement un test phénotypique de sensibilité aux antineuminidases sera réalisé en cas de positivité de la culture virale à J0 et J2.

Les souches virales seront conservées pendant 10 ans. Elles pourront faire, en cas de besoin, l’objet d’analyses complémentaires ultérieures.

5.11 Administration des médicaments étudiés

Auto-administration par les patients pendant 5 jours. Les médecins investigateurs peuvent être amenés à prescrire un traitement symptomatique complémentaire à leurs patients ; ils restent libres de leurs prescriptions.

5.12 Recueil des autoquestionnaires

Les autoquestionnaires seront remplis par le patient deux fois par jour jusqu’à J5, puis chaque jour jusqu’à J7 avec un recueil des symptômes cliniques, de la tolérance, de l’observance de la prise des antiviraux et des traitements associés.

5.13 Suivi des patients et recueil des données

5.13.1 Visite à J2

Le suivi à J2 est assuré par une infirmière de recherche clinique au domicile du patient.

Lors de cette visite, après 4 prises de traitement (dans certains cas 5 prises en fonction du moment de l’inclusion dans la journée) et juste avant la prise suivante, un prélèvement naso-pharyngé sera réalisé à l’aide d’un kit de prélèvement virologique spécifique, commercialisé et prêt à l’emploi. Une fois réalisé, le prélèvement sera conditionné et envoyé au laboratoire de virologie selon les procédures déjà établies par les CNR de la grippe.

5.13.2 Visite à J7

Le suivi à J7 est assuré par une visite de contrôle au cabinet du médecin investigateur, avec un recueil des signes cliniques, des complications, de la prise d’antibiotiques, des hospitalisations et des arrêts de travail éventuels, de la tolérance, de la survenue de nouveaux cas de syndromes grippaux dans le foyer et récupération des autoquestionnaires et des doses de traitement restantes.

5.13.3 Visite à J14

Le suivi à J14 est assuré par un contact téléphonique par une infirmière de recherche clinique avec un recueil de l’évolution clinique, des complications, de la prise d’antibiotiques, des arrêt de travail éventuels, du nombre de cas secondaires de grippe survenus dans le foyer du patient depuis l’inclusion et recueil de la tolérance.

5.14 Schéma de la prise des traitements étudiés, des prélèvements et des consultations

**Si consultation avant 18h : 2 prises le premier jour**															
	**J0 (inclusion)**	**J1 (2ème jour)**	**J2 (3ème jour)**	**J3 (4ème jour)**	**J4 (5ème jour)**	**J5**	**J6**	**J7**	**J8**	**J9**	**J10**	**J11**	**J12**	**J13**	**J14**
**Matin**			***Prélèvement (avant la 5ème prise)***					***Consultation médecin***							***Appel infirmière***
	1ère prise (lors de la visite d'inclusion)	3ème prise	5ème prise	7ème prise	9ème prise										
**Soir**															
	2ème prise	4ème prise	6ème prise	8ème prise	10ème prise (fin du traitement)										

**Si consultation après 18 h : 1 prise le premier jour**															
	**J0 (inclusion)**	**J1 (2ème jour)**	**J2 (3ème jour)**	**J3 (4ème jour)**	**J4 (5ème jour)**	**J5**	**J6**	**J7**	**J8**	**J9**	**J10**	**J11**	**J12**	**J13**	**J14**
**Matin**								***Consultation médecin***							***Appel infirmière***
		2ème prise	4ème prise	6ème prise	8ème prise										
**Soir**			***Prélèvement (avant la 5ème prise)***												
	1ère prise (lors de la visite d'inclusion)	3ème prise	5ème prise	7ème prise	9ème prise										

5.15 Liste des données recueillies

5.15.1 Recueil à J0 par le médecin investigateur

- Critères d'éligibilité, résultat du test de diagnostic rapide de la grippe
- Données cliniques :date de la visite, âge du patient, sexe, température par thermomètre digital fourni au patient à cette occasion (par voie buccale) et date d'apparition du constat ou du ressenti fébrile ; symptômes cliniques sur une échelle cotée 0 (= absent), 1 (= minime), 2 (= modérée), 3 (= important), et date d'apparition des symptômes suivants: premier symptôme, toux, douleur pharyngée, rhinorrhée, otite, céphalée, asthénie, myalgie, frissons, sensation de malaise. Recueil sur une échelle visuelle analogique validée de 10 points des capacités à effectuer des activités normales, de l'état général, de la qualité du sommeil. Recueil des caractéristiques du foyer à l'inclusion : nombre, âges, pour chaque sujet : syndrome grippal dans les 15 jours précédant l'inclusion (oui/non), vaccinations (oui/non) et traitements par inhibiteurs de neuraminidase (non/oui/nom de l'inhibiteur si oui et date de début/fin). Traitements prescrits. Signes de complications, hospitalisation et arrêt de travail.
- Recueil de l'heure de la première prise du traitement (la première prise est administrée en présence du médecin investigateur).

5.15.2 Recueil J0 à J7 par le patient : autoquestionnaire remis au patient par son médecin généraliste investigateur et rendu à ce dernier à J7

- Auto évaluation des symptômes cliniques (toux, obstruction nasale, douleur pharyngée, asthénie, céphalée, myalgies, sensation fébrile) par le patient deux fois par jour pendant les 5 jours de traitement, puis une fois par jour jusqu'à J7, sur une échelle cotée 0 (= absent), 1 (= minime), 2 (= modérée), 3 (= important) ; recueil sur une échelle visuelle analogique validée (12) de 10 points des capacités à effectuer des activités normales, de l'état général, de la qualité du sommeil. Enregistrement d'autres signes évocateurs d'une complication
- Recueil sur le carnet des prises de médicaments (ceux de l'essai, ceux prescrits, ceux en automédication)
- Recueil de l'observance rapportée (auto-évaluée avec la grille APROCO, Carrieri et al. JAIDS 2001 ; 28 :232-39)
- Evénements indésirables auto-rapportés par le patient
- A J0 uniquement : Activité professionnelle oui /non, profession, salarié/libéral oui/non, temps plein/partiel,

5.15.3 Recueil à J7 par le médecin investigateur

- Date de la visite, signes cliniques (les mêmes que ceux de J0), signes de complications, prise d'antibiotiques, hospitalisation et arrêt de travail.
- Evénements indésirables rapportés et/ou constatés à partir d'une liste pré-établie des évènements indésirables attendus, à partir des connaissances de pharmacovigilance concernant les produits utilisés. Les évènements indésirables inattendus seront systématiquement recherchés.
- Pour les sujets vivants dans des foyers de taille supérieure à 1, recueil de la survenue de cas de syndromes grippaux (fièvre de plus de 37°8 + toux ou mal de gorge) chez les

sujets contacts, et si oui, des dates de survenues des premiers symptômes et de la prise de traitement par inhibiteur de neuraminidase (non/oui/nom de l'inhibiteur si oui et date de début/fin).

5.15.4 Appel téléphonique à J14 par une infirmière de recherche clinique

En vue de cet appel téléphonique, le médecin recueillera le numéro de téléphone de l'endroit où se trouvera le patient à la date d'appel prévu, sur une fiche qui sera communiquée à l'infirmière de recherche clinique et ne fera pas l'objet d'une saisie informatisée. La date de l'appel sera enregistrée (J14 +/- 2)

Au cours de cet appel, l'infirmière :

- recueillera l'évolution clinique, les complications, la prise d'antibiotiques, et les arrêts de travail éventuels
- notera les événements indésirables rapportés et/ou constatés à partir d'une liste pré-établie d'évènements indésirables attendus, à partir des connaissances de pharmacovigilance concernant les produits utilisés. Les évènements indésirables inattendus seront systématiquement recherchés.
- pour les sujets vivants dans des foyers de taille supérieure à 1, recueillera la survenue de cas de syndromes grippaux (fièvre de plus de 37°8 + toux ou mal de gorge) entre J7 et J14, et si oui, des dates de survenues des premiers symptômes et de la prise de traitement par inhibiteur de neuraminidase (non/oui/nom de l'inhibiteur si oui et date de début/fin).

6 ANALYSES STATISTIQUES ET ECONOMIQUES

6.1 Méthodes d'analyse statistique

Les analyses statistiques seront réalisées au moyen des logiciels SAS et/ou R sous la responsabilité du Pr. France Mentré, au sein de l'UF de Biostatistiques de l'Hôpital Bichat, APHP. Les statistiques descriptives usuelles seront réalisées sur toutes les variables globalement et par bras de traitement.

6.1.1 Comparabilité des groupes

La comparabilité des groupes sera vérifiée pour l'ensemble des variables recueillies par le médecin et auto-recueillies par le patient à J0, ainsi que pour les données virologiques (tests rapides, mais aussi résultats de la RT-PCR, du type/sous-type/variant identifié par RT-PCR ou par isolement viral).

6.1.2 Analyse du critère principal

L'analyse principale sera réalisée en intention de traiter sur l'ensemble de la population des patients ayant une grippe de type A suspectée sur un test rapide positif, et ayant au moins reçu une dose d'antiviral. Deux comparaisons seront effectuées : l'une entre le bras 1 (bithérapie) et le bras 2 (oseltamivir + placebo) et une entre le bras 1 (bithérapie) et le bras 3 (zanimivir+ placebo), le risque de première espèce de chacun de ces tests sera de 0,025 afin de préserver une erreur globale de 0,05. Les proportions de patients ayant une RT-PCR négative à J2 entre les deux bras seront comparées par régression logistique avec ajustement sur le délai entre apparition des symptômes et prise du traitement (moins au plus de 24 heures). En cas de résultat virologique manquant à J2, le prélèvement sera considéré comme positif (échec).

Deux analyses complémentaires seront réalisées, l'une en intention de traiter sur la population des patients randomisés ayant une grippe clinique A virologiquement confirmée par RT-PCR, l'autre per protocole, sur la population des patients randomisés ayant une grippe clinique A virologiquement confirmée par RT-PCR et ayant reçu l'ensemble du traitement prévu avant le prélèvement à J2.

6.1.3 Analyse des critères secondaires

Pour les critères secondaires, deux comparaisons seront effectuées l'une entre le bras 1 (bithérapie) et le bras 2 (oseltamivir + placebo) et une entre le bras 1 (bithérapie) et le bras 3 (zanimivir + placebo). Le risque de première espèce ne sera pas corrigé et sera de 0,05 pour chaque test.

L'analyse sur les critères secondaires sera réalisée par régression logistique pour les variables qualitatives et de Wilcoxon modifié pour tenir compte de l'ajustement comme pour le critère principal.

Analyse des taux d'attaque secondaires dans les foyers par une équipe spécifique : pour les sujets vivants en foyer de taille supérieure à 1 (70% des inclusions), la proportion de cas secondaires survenant à J7 (et jusque J14) parmi l'ensemble des sujets n'ayant pas fait de grippe au moment de l'inclusion du sujet index sera comparé entre les bras. L'analyse tiendra compte de l'effet grappe du foyer, du délai entre les premiers symptômes du sujet inclus dans l'essai et la première prise de traitement, de l'existence de cas de syndromes grippaux antérieurement à l'inclusion, du statut vaccinal des sujets. Les méthodes utilisées seront les modèles d'équations estimantes généralisées en utilisant une corrélation échangeable (soit régression logistique alternante, soit

modèle pour données censurées). Une estimation par modélisation « Susceptible Infectious Recover » - SIR telle que décrite dans Cauchemez S, Carrat F, Viboud C, et al. Stat Med 2004, 23 :3469-87 sera réalisée.

A partir des données de l'essai, une analyse de type coût-efficacité sera conduite par une équipe spécifique, dont l'objectif sera d'évaluer l'impact médico-économique. Les coûts directs médicaux et les coûts indirects (i.e. le nombre et la durée des arrêts de travail) seront évalués pour chaque patient. Concernant la mesure des quantités physiques consommées, ces informations seront recueillies dans les cahiers d'observations (jours avec symptômes, nombre de symptômes, nombre et jours avec complications, effets secondaires, hospitalisations et durées, médicaments antiviraux et traitements concomitants, charge virale, autres examens, etc.). Les informations concernant les arrêts de travail seront également collectées au cours de l'essai via l'auto-questionnaire. Chaque composante des ressources consommées sera mesurée puis valorisée pour en déterminer le coût. Les coûts seront valorisés à partir des remboursements de la Sécurité Sociale. Le coût des traitements sera déterminé à partir des prix de catalogue des laboratoires pharmaceutiques. Les coûts indirects seront évalués sur la base des statistiques nationales sur le revenu des ménages. Le coût et l'efficacité estimés pour chaque groupe de traitement sur une période de 15 jours après inclusion seront comparés sous la forme du calcul d'un ratio coût-efficacité, permettant d'obtenir le coût (ou le gain) induit par le traitement expérimental (association oseltamivir et zanamivir) par unité d'efficacité supplémentaire, comparé au deux autres bras. Nous étudierons l'influence d'une variation de chaque paramètre de coûts et plus particulièrement du prix des antiviraux au travers d'une analyse de sensibilité. La variabilité de l'ensemble des paramètres cliniques et économiques sera prise en compte par la définition d'un intervalle de confiance des ratios coût-efficacité.
L'objectif de cette analyse économique est de déterminer la(les) stratégie(s) opérationnelle(s) pour le traitement de la grippe de type A et répondre ainsi à la question du « Comment traiter ? ». Aussi, dans un premier temps, nous retiendrons le critère d'efficacité principal de l'essai, à savoir la réponse virologique à court terme. Une première analyse coût-efficacité déterminera le ratio coût-efficacité de la bithérapie comparé à la monothérapie en terme de coût par RT-PCR négative additionnelle. Cependant, pour pouvoir comparer les résultats de l'analyse économique avec ceux d'études coût-efficacité déjà publiées sur la grippe, un critère secondaire sera estimé pour l'analyse économique : le coût additionnel par jour sans symptômes supplémentaires.
Une autre approche sera également envisagée, pour déterminer le rapport coût-bénéfice du traitement de la grippe A. Les bénéfices du traitement par antiviraux seront estimés sur la base du nombre de jours d'arrêt de travail évités et valorisés en termes monétaires. Les coûts du traitement antiviral et les bénéfices associés seront comparés sous la forme d'un bénéfice net.
Une dernière approche sera proposée et reposera sur l'analyse des cas secondaires (i.e. contagions évitées au sein du foyer). Une analyse de type coût-bénéfice sera réalisée. L'ensemble des bénéfices pour la société seront estimés. Ils comprendront les arrêts de travail évités et les contagions évitées, tous deux valorisés monétairement.

6.2 Justification du nombre de sujets

Un effectif de 300 sujets par bras de randomisation, soit au total 900 sujets remplissant les critères d'inclusion donc ayant un résultat positif pour le test de détection rapide pour le virus Influenza de type A sera nécessaire pour avoir une puissance de 90% de mettre en évidence une différence de proportion de RT-PCR négative entre la bithérapie et chaque monothérapie avec un seuil de signification de 0,025 pour prendre en compte les deux tests bilatéraux. Ce calcul repose sur l'hypothèse que la proportion de RT-PCR négative à J2 chez l'adulte après la prise de

la quatrième dose est de 55% sous monothérapie et qu'elle est augmentée de 15%, soit un total de 70%, sous bithérapie. Cette estimation de 55% repose sur les données de la littérature (Treanor *Jama 2000* (15), Nicholson *Lancet 2000* (12), Hayden *NEJM 1997* (7) et notamment dans Puhakka *Scand J Infect Dis 2003* : 56% de négativation de la RT-PCR dans le bras zanamivir versus 29% dans le bras placebo à 48h (p<0,001)). Ce calcul conduit au besoin d'inclusion de 270 patients par bras, nombre qui a été augmenté de 10% pour prendre en compte l'ajustement avec le délai (soit 300 patients). Il faut noter que cet effectif aura aussi une puissance de plus de 90% pour mettre en évidence une différence de 1 jour de durée des symptômes cliniques entre le bras bithérapie par rapport à chaque bras monothérapie (pour un écart-type de 3 jours). Dans la mesure où, sur un groupe de patients suspects de grippe en période épidémique, 60 à 70% ont un test diagnostic rapide positif, il est prévu de proposer ce test rapide de détection des virus Influenza de type A auprès de 1450 patients éligibles pour participer à l'essai.

Concernant l'analyse des cas secondaires, les hypothèses sont les suivantes : 70% des sujets inclus dans l'essai vivant en foyer ; une taille moyenne des foyers de taille supérieure à 1 de 3.1 sujets (données INSEE). On attend donc dans l'essai et par bras : 200 sujets inclus vivant en foyer et 420 sujets contacts. Parmi les sujets contacts on suppose que 3/4 n'ont pas présentés de syndrome grippaux à la date d'inclusion du sujet inclus et donc, 315 contacts seraient éligibles par bras pour le calcul du critère secondaire. En supposant un taux d'attaque secondaire de 12% à 15% en cas de monothérapie (Welliver R et al. JAMA 2001 ;285 :748 ; Hayden F et al. New Engl J Med 2000 ;343 :1282), l'essai aurait une puissance supérieure à 80% pour la mise en évidence d'une différence de 7% entre la bithérapie et la monothérapie. Un travail récent de réanalyse de 4 essais utilisant les inhibiteurs de neuraminidase (Halloran ME et al., Am J Epidemiol 2007 ;185 :212) a montré une réduction de la contagiosité des sujets traités par oseltamivir (80% (95%CI 43-93)) nettement supérieure à celle des sujets traités par zanamivir (19% (95%CI(-160-75)). L'étude aura une puissance supérieure à 99% pour cette comparaison.

6.3 Extrapolation des conclusions

L'essai se déroule en médecine de ville ; les inclusions sont réalisées par des médecins généralistes investigateurs qui pratiquent eux-mêmes les tests diagnostiques rapides. Les patients tout-venant, hommes et femmes, seront assez voisins des patients cibles d'une éventuelle indication d'une bi-thérapie à l'avenir. Nous pensons aussi que nos résultats pourraient dans une certaine mesure être extrapolés aux autres virus grippaux, si en cas de pandémie le bénéfice d'une bi-thérapie s'avérait intéressant pour le contrôle de l'extension pandémique ou si le développement rapide de résistances au cours de la première vague pandémique s'avérait de nature à compromettre l'usage d'une monothérapie antivirale. L'extrapolation à un virus pandémique - par définition inconnu - de l'efficacité et de la tolérance d'une association d'antiviraux mise en évidence pour les virus influenza de type A (H3N2) ou (H1N1), saisonniers, tient évidemment du pari. Néanmoins, en pareille circonstance, il sera préférable d'avoir une expérience précédente rigoureusement menée vis-à-vis d'une telle association plutôt que de ne disposer d'aucune information sur une telle stratégie.

7 EVALUATION DE LA SECURITE

7.1 Description des paramètres d'évaluation de la sécurité

- **Evènement indésirable**
  Toute manifestation nocive survenant chez une personne qui se prête à une recherche biomédicale que cette manifestation soit liée ou non à la recherche ou au produit sur lequel porte cette recherche.

- **Effet indésirable d'un médicament expérimental**
  Toute réaction nocive et non désirée à un médicament expérimental quelle que soit la dose administrée

- **Evènement ou effet indésirable grave**
  Tout évènement ou effet indésirable qui entraîne la mort, met en danger la vie de la personne qui se prête à la recherche, nécessite une hospitalisation ou la prolongation de l'hospitalisation, provoque une incapacité ou un handicap importants ou durables, ou bien se traduit par une anomalie ou une malformation congénitale, et s'agissant du médicament, quelle que soit la dose administrée.

- **Effet indésirable inattendu d'un médicament expérimental**
  Tout effet indésirable dont la nature, la sévérité ou l'évolution ne concorde pas avec les informations figurant dans le résumé des caractéristiques du produit lorsque le médicament est autorisé, et dans la brochure pour l'investigateur lorsqu'il n'est pas autorisé.

- **Fait nouveau**
  Toute nouvelle donnée de sécurité, pouvant conduire à une réévaluation du rapport des bénéfices et des risques de la recherche ou du médicament expérimental, ou qui pourrait être suffisant pour envisager des modifications dans l'administration du médicament expérimental, dans la conduite de la recherche.

7.2 Méthodes et calendrier prévus pour mesurer, recueillir et analyser les paramètres d'évaluation de la sécurité

7.2.1 Comité de pilotage

Il sera constitué des initiateurs cliniciens du projet, du biostatisticien en charge du projet, des représentants du promoteur et de l'URC nommés pour cette recherche.

Il définira l'organisation générale et le déroulement de la recherche et coordonnera les informations.

Il déterminera initialement la méthodologie et décidera en cours de recherche des conduites à tenir dans les cas imprévus, surveillera le déroulement de la recherche en particulier sur le plan de la tolérance et des évènements indésirables.

7.2.2 Comité de vigilance

Il a une fonction consultative lorsque le promoteur fait appel à lui sur des points médicaux tels la tolérance et les événements indésirables.

7.3 Procédures mises en place en vue de l'enregistrement et de la notification des évènements indésirables

7.3.1 Evènements indésirables non graves

Tout événement indésirable - non grave suivant la définition précédente - observé lors de la recherche et dans ses suites devra être reporté dans le cahier d'observation dans la section prévue à cet effet.

Un seul évènement doit être reporté par item. L'évènement peut correspondre à un symptôme, un diagnostic ou à un résultat d'examen complémentaire jugé significatif. Tous les éléments cliniques ou para-cliniques permettant de décrire au mieux l'évènement correspondant doivent être reportés.

7.3.2 Evènements indésirables graves (EIG)

Les investigateurs doivent notifier immédiatement au promoteur AP-HP les évènements indésirables graves tels que définis ci-dessus.

L'investigateur complète les formulaires d'évènements indésirables graves (du cahier d'observation de la recherche) et les envoie au DRCD par fax au 01 44 84 17 99 et ce, dans les 48 heures (après si possible un appel téléphonique immédiat au 01 44 84 17 23 en cas de décès ou d'une menace vitale).

L'investigateur doit également informer l'URC en charge de la recherche de la survenue de l'EIG.

Pour chaque évènement indésirable grave, l'investigateur devra émettre un avis sur le lien de causalité de l'évènement avec chaque médicament expérimental et les autres traitements éventuels.

L'obtention d'informations relatives à la description et l'évaluation d'un évènement indésirable peuvent ne pas être possibles dans le temps imparti pour la déclaration initiale.

Aussi, l'évolution clinique ainsi que les résultats des éventuels bilans cliniques et des examens diagnostiques et/ou de laboratoire, ou toute autre information permettant une analyse adéquate du lien de causalité seront rapportés :

- soit sur la déclaration initiale d'EIG s'ils sont immédiatement disponibles,
- soit ultérieurement et le plus rapidement possible, en envoyant par fax une nouvelle déclaration d'EIG complétée (et en précisant qu'il s'agit d'un suivi d'EIG déclaré et le numéro de suivi).

Toutes les déclarations faites par les investigateurs devront identifier chaque sujet participant à la recherche par un numéro de code unique attribué à chacun d'entre eux.

En cas de décès notifié d'un sujet participant à la recherche, l'investigateur communiquera au promoteur tous les renseignements complémentaires demandés (compte-rendu d'hospitalisation, résultats d'autopsie…).

Tout fait nouveau survenu dans la recherche ou dans le contexte de la recherche, provenant de données de la littérature ou de recherches en cours, devra être notifié au promoteur.

Déclaration des évènements indésirables graves aux Autorités de Santé

Elle sera assurée par le Pôle de Pharmacovigilance du DRCD, après évaluation de la gravité de l'évènement indésirable, du lien de causalité avec chaque médicament expérimental et les autres traitements éventuels ainsi que du caractère inattendu des effets indésirables.

Toutes les suspicions d'effet indésirable grave inattendu seront déclarées par le promoteur aux autorités compétentes dans les délais légaux.

Toute donnée de sécurité ou tout fait nouveau qui pourrait modifier significativement l'évaluation du rapport des bénéfices et des risques d'un médicament expérimental, ou de la recherche, ou qui pourrait conduire à envisager des modifications concernant l'administration du médicament ou la conduite de la recherche, sera transmise par le promoteur aux autorités compétentes, au Comité de Protection des Personnes et aux investigateurs de la recherche. Par exemple :

a) toute augmentation cliniquement significative de la fréquence d'apparition d'un effet indésirable grave attendu ;

b) des suspicions d'effet indésirable grave inattendu survenus chez des participants ayant terminé l'essai et qui sont notifiés par l'investigateur au promoteur, ainsi que des rapports de suivi éventuels ;

c) tout fait nouveau concernant le déroulement de l'essai clinique ou le développement du médicament, lorsque ce fait nouveau est susceptible de porter atteinte à la sécurité des participants. A titre d'exemple :

- un événement indésirable grave susceptible d'être lié aux investigations et aux procédures de diagnostic de l'essai et qui pourrait modifier le déroulement de cet essai,
- un risque significatif pour la population de l'essai comme par exemple un manque d'efficacité du médicament utilisé dans le traitement d'une maladie mettant en jeu le pronostic vital,
- des résultats significatifs de sécurité issus d'une étude menée chez l'animal récemment terminée (telle qu'une étude de carcinogénicité),
- un arrêt anticipé ou une interruption temporaire pour des raisons de sécurité d'un essai conduit avec le même médicament dans un autre pays,
- un effet indésirable grave inattendu lié à un médicament non expérimental nécessaire à la réalisation de l'essai (ex : « challenge agents », traitement de secours)

d) tout effet indésirable grave inattendu transmis au promoteur par un autre promoteur d'un essai clinique mené dans un pays tiers portant sur le même médicament.

7.4 Modalités et durée du suivi des personnes suite à la survenue d'évènements indésirables

Tout patient présentant un évènement indésirable doit être suivi jusqu'à la résolution ou la stabilisation de celui-ci.

- Si l'évènement n'est pas grave, l'évolution en sera notée sur la page correspondante du cahier d'observation à la section prévue à cet effet.
- Si l'évènement est grave, un suivi d'EIG sera envoyé au DRCD.

Le recueil et le traitement informatique de ces informations entrent dans le cadre des lois n°78-17 du 6 janvier 1978 et n°94-548 du 1er juillet 1994 (dites "Informatique et Libertés"). Les patients reçoivent une information détaillée quant à la nature des données recueillies, leur utilisation prévue, l'objectif de l'essai, les destinataires des données, son droit d'accès et de rectification, ainsi que son droit d'opposition ou d’arrêt de l’essai sans justification à fournir.

Seuls sont inclus les patients ayant accepté de donner leur consentement exprès écrit et éclairé préalable, après avoir pu lire une lettre d'information qui leur a été expliquée.

Le traitement des données sera soumis à une autorisation de la Commission Informatique et Libertés (CNIL), après un avis favorable du Comité Consultatif sur le Traitement de l'Information en matière de Recherche dans le domaine de la Santé (CCTIRS). Les termes de cette autorisation apparaîtront dans la lettre d'information et le recueil de consentement.

8 DUREE DE L'ETUDE ET CALENDRIER

Les inclusions se dérouleront durant la période correspondant à l'épidémie de grippe saisonnière durant l'hiver 2008/2009, si l'ensemble des financements, de l'approvisionnement des investigateurs en médicaments actifs et placebo et des accords réglementaires ont été obtenus.

- Les données seront saisies en temps réel et la base de données gelée dès le printemps 2009.
- L'analyse et le rapport final seront remis à l'automne 2009.

Si les financements et/ou les accords réglementaires n'étaient pas obtenus au moment du démarrage de l'épidémie de grippe de la saison 2008-2009, l'étude serait repoussée d'une année.

Si le recrutement des patients durant la période d'inclusion prévue ci-dessus est insuffisant, l'étude pourra être prolongée au cours de la période épidémique de l'hiver suivant.

9 DROIT D'ACCES AUX DONNEES ET DOCUMENTS SOURCE

Les personnes ayant un accès direct conformément aux dispositions législatives et réglementaires en vigueur, notamment les articles L.1121-3 et R.5121-13 du code de la santé publique (par exemple, les investigateurs, les personnes chargées du contrôle de qualité, les moniteurs, les assistants de recherche clinique, les auditeurs et toutes personnes appelées à collaborer aux essais) prennent toutes les précautions nécessaires en vue d'assurer la confidentialité des informations relatives aux médicaments expérimentaux, aux essais, aux personnes qui s'y prêtent et notamment en ce qui concerne leur identité ainsi qu’aux résultats obtenus. Les données collectées par ces personnes au cours des contrôles de qualité ou des audits sont alors rendues anonymes.

10 CONTROLE ET ASSURANCE DE LA QUALITE

La recherche sera encadrée selon les procédures opératoires standard du promoteur.
Le déroulement de la recherche dans les centres investigateurs et la prise en charge des sujets sera faite conformément à la déclaration d'Helsinki et les Bonnes Pratiques en vigueur.

10.1 Procédures de monitoring

Les ARC représentants du promoteur (le monitorage sera effectué par une CRO) effectueront des visites des centres investigateurs au rythme correspondant au schéma de suivi des patients dans le protocole, aux inclusions dans les différents centres et au niveau de risque D qui a été attribué à la recherche (100% des données seront vérifiées).

- Visite d'ouverture de chaque centre : avant inclusion, pour une mise en place du protocole et prise de connaissance avec les différents intervenants de la recherche biomédicale.
- Lors des visites suivantes, les cahiers d'observation seront revus au fur et à mesure de l'état d'avancement de la recherche par les ARC. L'investigateur principal de chaque centre ainsi que les autres investigateurs qui incluent ou assurent le suivi des personnes participant à la recherche s'engagent à recevoir les ARC à intervalles réguliers.

Lors de ces visites sur site et en accord avec les Bonnes Pratiques Cliniques, les éléments suivants seront revus :

- Respect du protocole et des procédures définies pour la recherche,
- Vérification des consentements éclairés des patients
- Examen des documents source et confrontation avec les données reportées dans le cahier d'observation quant à l'exactitude, les données manquantes, la cohérence des données selon les règles édictées par les procédures du DRCD.

Visite de fermeture : récupération des cahiers d'observation, bilan à la pharmacie, documents de la recherche biomédicale, archivage.

10.2 Transcription des données dans le cahier d'observation

Toutes les informations requises par le protocole doivent être fournies dans le cahier d'observation et une explication donnée par l'investigateur pour chaque donnée manquante.
Les données devront être transférées dans les cahiers d'observation au fur et à mesure qu'elles sont obtenues qu'il s'agisse de données cliniques ou para-cliniques. Les données devront être copiées de façon nette et lisible à l'encre noire dans ces cahiers (ceci afin de faciliter la duplication et la saisie informatique).
Les données erronées dépistées sur les cahiers d'observation seront clairement barrées et les nouvelles données seront copiées sur le cahier avec les initiales et la date par le membre de l'équipe de l'investigateur qui aura fait la correction.
L'anonymat des sujets sera assuré par un numéro de code et les initiales de la personne qui se prête à la recherche sur tous les documents nécessaires à la recherche, ou par effacement par les moyens appropriés des données nominatives sur les copies des documents source, destinés à la documentation de la recherche.
Les données informatisées sur un fichier seront déclarées à la CNIL selon la procédure adaptée au cas.

11 CONSIDERATIONS LEGALES ET ETHIQUES

Le promoteur est défini par la loi 2004-806 du 9 août 2004. Dans cette recherche, l'AP-HP est le promoteur et le Département de la Recherche Clinique et du Développement (DRCD) en assure les missions réglementaires.

Avant de démarrer la recherche, chaque investigateur fournira au représentant du promoteur de la recherche une copie de son **curriculum vitæ personnel daté et signé** et comportant son numéro d'inscription à l'Ordre des Médecins.

11.1 Demande d'autorisation auprès de l'Afssaps

Pour pouvoir démarrer la recherche, l'AP-HP en tant que promoteur doit soumettre un dossier de demande d'autorisation auprès de l'autorité compétente l'Afssaps. L'autorité compétente, définie à l'article L. 1123-12, se prononce au regard de la sécurité des personnes qui se prêtent à une recherche biomédicale, en considérant notamment la sécurité et la qualité des produits utilisés au cours de la recherche conformément, le cas échéant, aux référentiels en vigueur, leur condition d'utilisation et la sécurité des personnes au regard des actes pratiqués et des méthodes utilisées ainsi que les modalités prévues pour le suivi des personnes.

11.2 Demande d'avis au Comité de Protection des Personnes

En accord avec l'article L.1123-6 du Code de Santé Publique, le protocole de recherche doit être soumis par le promoteur à un Comité de Protection des Personnes L'avis de ce comité est notifié à l'autorité compétente par le promoteur avant le démarrage de la recherche.

11.3 Modifications

Le DRCD doit être informé de tout projet de modification du protocole par l'investigateur coordonnateur.
Les modifications devront être qualifiées en substantielles ou non.

Une modification substantielle est une modification susceptible, d'une manière ou d'une autre, de modifier les garanties apportées aux personnes qui se prêtent à la recherche biomédicale (modification d'un critère d'inclusion, prolongation d'une durée d'inclusion, participation de nouveaux centres,…).

Après le commencement de la recherche, toute modification substantielle de celle-ci à l'initiative du promoteur doit obtenir, préalablement à sa mise en oeuvre, un avis favorable du comité et une autorisation de l'autorité compétente. Dans ce cas, si cela est nécessaire, le comité s'assure qu'un nouveau consentement des personnes participant à la recherche est bien recueilli.

Par ailleurs, toute extension de la recherche (modification profonde du schéma thérapeutique ou des populations incluses, prolongation des traitements et ou des actes thérapeutiques non prévus initialement dans le protocole) devra être considérée comme une nouvelle recherche.
Toute modification substantielle devra faire l'objet par le promoteur après paiement d'une taxe d'une demande d'autorisation auprès de l'Afssaps et/ou d'une demande d'avis du CPP.

11.4 Déclaration CNIL

La loi prévoit que la déclaration du fichier informatisé des données personnelles collectées pour la recherche doit être faite avant le début effectif de la recherche.

Une méthodologie de référence spécifique au traitement de données personnelles opéré dans le cadre des recherches biomédicales définies par la loi 2004-806 du 9 août 2004 car entrant dans le champ des articles L.1121-1 et suivants du Code de Santé Publique a été établie par la CNIL en janvier 2006.

Cette méthodologie permet une procédure de déclaration simplifiée lorsque la nature des données recueillies dans la recherche est compatible avec la liste prévue par la CNIL dans son document de référence.

Lorsque le protocole bénéficie d'un contrôle qualité des données par un ARC représentant le promoteur et qu'il entre dans le champ d'application de la procédure simplifiée CNIL, le DRCD en qualité de promoteur demandera au responsable du fichier informatique de s'engager par écrit sur le respect de la méthodologie de référence MR06001 simplifiée.

11.5 Note d'information et Consentement éclairé

Voir en annexe.

11.6 Rapport final de la recherche

Le rapport final de la recherche sera écrit en collaboration par le coordonnateur et le biostatisticien pour cette recherche. Ce rapport sera soumis à chacun des investigateurs pour avis. Une fois qu'un consensus aura été obtenu, la version finale devra être avalisée par la signature de chacun des investigateurs et adressée au promoteur dans les meilleurs délais après la fin effective de la recherche. Un rapport rédigé selon le plan de référence de l'autorité compétente doit être transmis à l'autorité compétente ainsi qu'au CPP dans un délai de un an, après la fin de la recherche, s'entendant comme la dernière visite de suivi du dernier sujet inclus. Ce délai est rapporté à 90 jours en cas d'arrêt prématuré de la recherche.

12 TRAITEMENT DES DONNEES ET CONSERVATION DES DOCUMENTS ET DES DONNEES RELATIVES A LA RECHERCHE

Les documents d'une recherche entrant dans le cadre de la loi sur les recherches biomédicales doivent être archivés par toutes les parties pendant une durée de 15 ans après la fin de la recherche.

(voir BPC, chapitre 8 : documents essentiels)

Cet archivage indexé comporte :

- Les copies de courrier d'autorisation de l'Afssaps et de l'avis obligatoire du CPP,
- Les versions successives du protocole (identifiées par le n° de version et la date de version),
- Les courriers de correspondance avec le promoteur,
- Les consentements signés des sujets sous pli cacheté avec la liste ou registre d'inclusion en correspondance,
- Le cahier d'observation complété et validé de chaque sujet inclus,
- Toutes les annexes spécifiques à l'étude,
- Le rapport final de l'étude provenant de l'analyse statistique et du contrôle qualité de l'étude (double transmis au promoteur),
- Les certificats d'audit éventuels réalisés au cours de la recherche.

La base de données ayant donné lieu à l'analyse statistique doit aussi faire l'objet d'archivage par le responsable de l'analyse (support papier ou informatique).

13 ASSURANCE ET ENGAGEMENT SCIENTIFIQUE

13.1 Assurance

L'Assistance Publique- Hôpitaux de Paris est le promoteur de cette recherche. En accord avec la loi sur les recherches biomédicales, elle a pris une assurance auprès de la compagnie GERLING KONZERN pour toute la durée de la recherche, garantissant sa propre responsabilité civile ainsi que celle de tout intervenant (médecin ou personnel impliqué dans la réalisation de la recherche) (loi n°2004-806, Art L.1121-10 du CSP).

L'Assistance Publique - Hôpitaux de Paris se réserve le droit d'interrompre la recherche à tout moment pour des raisons médicales ou administratives; dans cette éventualité, une notification sera fournie à l'investigateur.

13.2 Engagement scientifique

Chaque investigateur s'engagera à respecter les obligations de la loi et à mener la recherche selon les B.P.C., en respectant les termes de la déclaration d'Helsinki en vigueur. Pour ce faire, un exemplaire de l'engagement scientifique (document type DRCD) daté et signé par chaque investigateur de chaque service clinique d'un centre participant sera remis au représentant du promoteur.

14 REGLES RELATIVES A LA PUBLICATION

L'AP-HP est propriétaire des données et aucune utilisation ou transmission à un tiers ne peut être effectuée sans son accord préalable.

Seront premiers signataires des publications, les personnes ayant réellement participé à l'élaboration du protocole et son déroulement ainsi qu'à la rédaction des résultats.

L'Assistance Publique- Hôpitaux de Paris doit être mentionnée comme étant le promoteur de la recherche biomédicale et comme soutien financier le cas échéant. les termes « Assistance Publique- Hôpitaux de Paris » doivent apparaître dans l'adresse des auteurs.

Les résultats de l'étude feront l'objet de publications dans des journaux scientifiques et de présentations lors de réunions scientifiques. Pour toute publication, l'investigateur principal élaborera à l'avance avec l'ensemble des investigateurs du Conseil scientifique des règles de valorisation dont il assurera l'arbitrage.

15 ANNEXES

15.1 Engagements du promoteur et de l'investigateur

- Le protocole a été revu et approuvé par le comité scientifique de l'essai et le sera par le promoteur avant sa mise en oeuvre. L'information contenue est conforme aux principes moraux, éthiques et scientifiques définis dans la déclaration d'Helsinki, et dans les lois et réglementations françaises.

- Les investigateurs seront informés de toutes les données nouvelles ou significatives, de tout élément nouveau concernant l'étude.

15.2 Cahier d'observation

15.2.1 Informations recueillies par le médecin généraliste ou l'infirmière investigateur

Visite de présélection à J0 (médecin investigateur)

- initiales du nom et du prénom, sexe, âge en année révolue,
- critères d'inclusion et d'exclusion

Inclusion et randomisation (médecin investigateur)

- résultat du test diagnostique rapide pour le virus Influenza de type A
- prélèvement
- bras de randomisation et numéro du patient dans l'essai (attribution automatique centralisée en ligne)
- check-list des documents, médicaments, et conseils remis au patient
- date de la visite
- température par thermomètre digital fourni au patient à cette occasion (par voie buccale) et date d'apparition du constat ou du ressenti fébrile ; symptômes cliniques sur une échelle cotée 0 (= absent), 1 (= minime), 2 (= modérée), 3 (= important), et date d'apparition des symptômes suivants: premier symptôme, toux, douleur pharyngée, rhinorrhée, otite, céphalée, asthénie, myalgie, frissons, sensation de malaise. Recueil sur une échelle visuelle analogique validée (12) de 10 points des capacités à effectuer des activités normales, de l'état général, de la qualité du sommeil.
- Recueil des caractéristiques du foyer à l'inclusion : nombre, âges, pour chaque sujet : syndrome grippal dans les 15 jours précédant l'inclusion (oui/non), vaccinations (oui/non) et traitements par inhibiteurs de neuraminidase (non/oui/nom de l'inhibiteur si oui et date de début/fin).
- Traitements prescrits. Signes de complications, hospitalisation et arrêt de travail.

- Recueil de l'heure de la première prise du traitement (la première prise est administrée en présence du médecin investigateur).

Visite ou consultation à J2 (infirmière de recherche clinique)

- prélèvement

Visite ou consultation à J7 (médecin investigateur)

- Date de la visite
- Signes cliniques (les mêmes que ceux de J0)
- Signes de complications, prise d'antibiotiques, hospitalisation et arrêt de travail
- Evénements indésirables rapportés et/ou constatés à partir d'une liste pré-établie des évènements indésirables attendus, à partir des connaissances de pharmacovigilance concernant les antiviraux utilisés. Les évènements indésirables inattendus y seront systématiquement recherchés.
- Pour les sujets vivants dans des foyers de taille supérieure à 1, recueil de la survenue de cas de syndromes grippaux (fièvre de plus de 37°8 + toux ou mal de gorge) chez les sujets contacts, et si oui, des dates de survenues des premiers symptômes et de la prise de traitement par inhibiteur de neuraminidase (non/oui/nom de l'inhibiteur si oui et date de début/fin).
- Evaluation de l'observance (boîtes)

Contact téléphonique à J14 (infirmière de recherche clinique)

- Date de l'appel
- Recueil de l'évolution clinique, des complications, de la prise d'antibiotiques, des arrêts de travail éventuels.
- Evénements indésirables rapportés et/ou constatés à partir d'une liste pré-établie des évènements indésirables attendus, à partir des connaissances de pharmacovigilance concernant les antiviraux utilisés. Les évènements indésirables inattendus y seront systématiquement recherchés.
- Pour les sujets vivants dans des foyers de taille supérieure à 1, recueil de la survenue de cas de syndromes grippaux (fièvre de plus de 37°8 + toux ou mal de gorge) entre J7 et J14, et si oui, des dates de survenues des premiers symptômes et de la prise de traitement par inhibiteur de neuraminidase (non/oui/nom de l'inhibiteur si oui et date de début/fin).

15.2.2 Informations recueillies par les virologues des deux CNR grippe (France Nord et France Sud)

Sous forme d'une fiche à remplir par le clinicien préleveur à chaque visite :

- Identité du préleveur

- Date, heure et lieu du prélèvement respiratoire

- Données démographiques du patient (numéro d'identification, DNN, sexe, profession, lieu de résidence)

- Identification du type de prélèvement: Prélèvement à l'inclusion ou en post inclusion ; difficultés liées à la réalisation du prélèvement-Notion d'épidémie familiale à l'inclusion

- Contexte clinique (signes à la phase d'état)

- Test rapide immunochromatographique EIA (positif pour la grippe A, négatif, douteux),

Au laboratoire de virologie (CNR grippe Nord et Sud) et de manière prospective :

- résultat de la RT-PCR quantitative pour le virus Influenza A (positif ou négatif).
- Isolement des souches virales de grippe A en culture cellulaire et détermination du sous-type et des variants.
- Pour les virus isolés en culture cellulaire à J0 et J2 réalisation des tests de sensibilité aux antineuraminidases (valeurs des IC50).

15.2.3 Informations recueillies par auto-questionnaire (patient)

Auto suivi biquotidien jusqu'à J5, puis quotidien jusqu'à J7 :

- Auto évaluation des symptômes cliniques (toux, obstruction nasale, douleur pharyngée, asthénie, céphalée, myalgies, sensation fébrile) sur une échelle cotée 0 (= absent), 1 (= minime), 2 (= modérée), 3 (= important)
- Recueil sur une échelle visuelle analogique validée (12) de 10 points des capacités à effectuer des activités normales, de l'état général, de la qualité du sommeil. Recueil des autres signes d'une complication
- Recueil sur le carnet des prises de médicaments (ceux de l'essai, ceux prescrits, ceux en automédication)
- Recueil de l'observance rapportée (auto-évaluée avec la grille APROCO, Carrieri et al. JAIDS 2001 ; 28 :232-39)
- Evénements indésirables auto-rapportés par le patient

15.3 Lettre d'information au patient

ASSISTANCE PUBLIQUE HÔPITAUX DE PARIS

FORMULAIRE D'INFORMATION

BIVIR : Essai randomisé en double aveugle comparant l'association oseltamivir et zanamivir à l'oseltamivir et placebo et au zanamivir et placebo dans le traitement curatif de la grippe virologiquement suspectée en médecine ambulatoire.

Le docteur ………… ……………………….., médecin investigateur m'a proposé de participer à une recherche biomédicale intitulée BIVIR «Essai randomisé en double aveugle comparant l'association oseltamivir et zanamivir à l'oseltamivir et placebo et au zanamivir et placebo dans le traitement curatif de la grippe virologiquement suspectée en médecine ambulatoire ». Cet essai s'inscrit dans le cadre d'une possible pandémie grippale dans les années à venir.

L'Assistance Publique - Hôpitaux de Paris est promoteur de cet essai. Le médecin m'a précisé que j'étais libre d'accepter ou de refuser de participer à cette recherche.

Avant que vous décidiez de participer à cette étude, il est important que vous lisiez attentivement et que vous compreniez cette notice d'information. N'hésitez pas à poser toutes les questions que vous voulez à votre médecin.

Il n'y a aucune obligation à participer à cette étude, c'est vous qui choisissez d'y participer. Après avoir lu cette notice d'information et avoir discuté de l'étude avec votre médecin, et si vous êtes d'accord pour y participer, vous devrez signer le formulaire de consentement qui confirme votre accord pour participer à cette recherche.

Pourquoi cette étude ?

La grippe est une infection saisonnière fréquente qui peut être responsable de signes cliniques marqués et intenses, empêchant de mener une activité normale et obligeant la personne à rester couchée. La durée moyenne des signes est d'environ 5 jours. Actuellement, le traitement de la grippe (de type A et B) nécessite la prescription d'un antiviral, soit le zanamivir soit l'oseltamivir, pendant une durée de 5 jours. Ce traitement diminue la durée des signes en moyenne d'une journée.

L'étude à laquelle nous vous proposons de participer a pour but de déterminer si l'association des ces 2 médicaments antiviraux (oseltamivir plus zanamivir) chez un même individu pendant 5 jours améliore l'efficacité du traitement contre la grippe, comparé à l'administration d'un seul antigrippal, sans majorer le risque d'effets secondaires.

L'efficacité du traitement sera évaluée par la mesure de la durée des signes ainsi que par la mesure de la durée pendant laquelle le virus continue à être présent dans l'organisme.

Quel traitement prendrez-vous ?

L'oseltamivir (Tamiflu®) est administré sous la forme d'1 comprimé à 75 mg matin et soir pendant 5 jours chez l'adulte. Le zanamivir (Relenza®) est inhalé lors d'une respiration forcée. Il s'administre à raison de 2 inhalations matin et soir pendant 5 jours. Ces deux traitements ont prouvé leur efficacité dans le traitement de la grippe en réduisant la durée des signes en rapport avec la maladie d'environ 1 à 2 jours, sur un total de 5 à 7 jours de maladie.

Si vous acceptez de participer à l'essai, vous recevrez après tirage au sort soit l'association oseltamivir plus zanamivir, soit l'oseltamivir associé à un placebo, soit le zanamivir associé à un placebo. Le placebo est une substance sans danger et sans activité pharmacologique.

Qui peut participer ?

Cette recherche biomédicale se déroulera au sein de cabinets de médecins généralistes sur toute la France et sur une durée globale de 10 semaines. 900 personnes participeront à cet essai, la durée de participation pour chaque participant étant de 14 jours.

Toute personne, présentant pendant la saison grippale des signes évocateurs de grippe depuis moins de 36 heures et pour lesquels un test rapide confirme la présence de virus grippal au niveau de son nez peut participer à cette étude. Les signes évocateurs de grippe sont la fièvre supérieure à 38°C (lors de la consultation, ou ressenti depuis 48 heures) avec au moins un signe respiratoire (toux, douleur de la gorge, écoulement nasal) et au moins un signe général (maux de tête, fatigue, courbatures, frissons, ou sensation de malaise). La personne devra être majeur et ne pas comporter de critères excluant sa participation à l'étude. Votre médecin précisera avec vous les critères listés ci-dessous :

Critères d'inclusion

- Période de grippe saisonnière déclarée au niveau national
- Patient présentant un syndrome grippal comportant: fièvre ≥ 38°C (lors de la consultation, ressenti ou déclaré par le patient) avec au moins un symptôme respiratoire (toux, douleur pharyngée, rhinorrhée) et au moins un symptôme général (céphalée, asthénie, myalgies, frissons, ou sensation de malaise)
- Patient âgé de plus de 18 ans des deux sexes
- Patient paraissant capable de comprendre et remplir l'autoquestionnaire qui lui sera remis à l'issue de la consultation
- Patient dont la symptomatologie grippale a débuté depuis moins de 36 heures. Le début des symptômes est défini comme le premier constat ou ressenti fébrile
- Patient bénéficiaire de la sécurité sociale ou de la CMU
- Patient ayant eu un examen médical préalable
- Patient ayant un test diagnostique rapide positif pour une infection grippale de type A
- Patient volontaire pour participer à l'essai, ayant lu la lettre d'information et donné son accord écrit de participation à l'essai

Critères de non inclusion

- Patient vacciné contre la grippe pour la saison épidémique en cours
- Patient incapable d'utiliser correctement l'inhalateur de zanamivir
- Patient asthmatique ou présentant une broncho-pneumopathie obstructive en poussée, ou ayant une maladie chronique sévère en poussée
- Patient ayant pris des antiviraux (oseltamivir, zanamivir ou amantadine) dans les 14 jours qui précèdent l'inclusion
- Patient ayant des antécédents de dépression ayant nécessité la prescription d'antidépresseur, convulsion ou trouble psychiatrique grave
- Femme enceinte confirmée par un test de grossesse (bandelette urinaire) ou allaitante
- Patient présentant une insuffisance rénale connue (clairance de la créatinine ≤ 30 ml/min)
- Patient présentant une hypersensibilité connue à l'un des principes actifs ou excipients

Schéma de l'étude

Le schéma suivant résume le plan expérimental de l'étude.

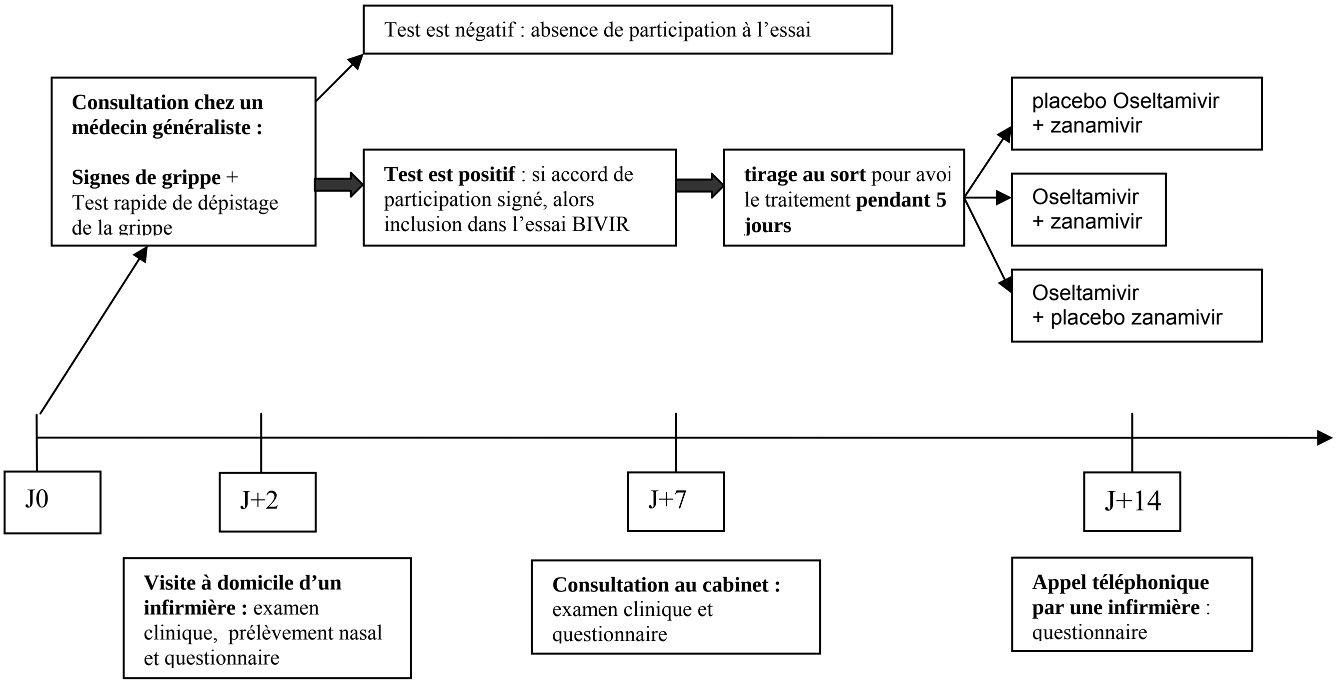

Comment se déroule l'étude ?

1) Visite d'inclusion dans l'essai :

Vous venez consulter votre médecin généraliste. Lors de cette visite, votre médecin observe un certain nombre de symptômes qui lui font suspecter la grippe (toux, fièvre, douleur à la gorge, maux de tête, fatigue, courbatures,…) et a pratiqué un test rapide de la grippe à partir d'un prélèvement de narine permettant d'affirmer que vous avez la grippe. A ce stade de la consultation, le médecin vous présente l'essai et demande votre accord pour y participer.

Après tirage au sort, vous recevrez soit 1/ l'association oseltamivir plus zanamivir, soit 2/ l'association oseltamivir plus placebo, soit 3/ l'association zanamivir plus placebo. Ni vous, ni votre médecin, ne saurez si vous recevez l'une ou l'autre de ces trois combinaisons. Un 2ème prélèvement au niveau de l'autre narine sera effectué afin de mettre en évidence le virus responsable de la grippe dont vous êtes atteint.

Votre médecin vous remettra les médicaments de l'essai en fonction des résultats du tirage au sort. En aucun cas vous ne devrez prendre de médicaments contre la grippe achetés dans une pharmacie ou que vous avez chez vous. Les médicaments antiviraux prescrits par votre médecin ne devront en aucune manière être donnés ou partagés avec quelqu'un d'autre. Les médicaments seront administrés pendant 5 jours (matin et soir). Vous débuterez le traitement au cabinet du médecin lors de la première consultation et votre médecin vous expliquera quand prendre votre traitement une fois rentré chez vous. Lors de cette première consultation, votre médecin vous fixera un rendez vous 7 jours plus tard.

2) Visites de suivi :

Au 2ème jour de traitement, une infirmière se déplacera à votre domicile. Au cours de cette visite gratuite, l'infirmière pratiquera à nouveau un prélèvement au niveau de l'une de vos 2 narines pour rechercher la persistance du virus. **Cette visite est capitale car c'est sur ce prélèvement que sera jugée l'efficacité des traitements.** Il faudra donc prendre vos disponibilités pour pouvoir recevoir de façon certaine l'infirmière.

Au 7ème jour, vous devrez revenir voir à son cabinet votre médecin généraliste. Cette consultation sera gratuite. Elle permettra d'évaluer la façon dont vous vous portez et dont vous aurez toléré les médicaments que vous avez reçus.

Pendant les 5 jours de traitement, matin et soir, et jusqu'à la consultation du 7ème jour, vous devrez remplir de façon très consciencieuse un questionnaire portant sur les signes en rapport avec la maladie (présence de fièvre, toux, courbatures …). Il y aura donc un questionnaire à remplir à chaque prise du traitement. Vous noterez également la prise des traitements antiviraux, ainsi que toute anomalie, ou signe clinique qui vous semblerait inhabituel.

Si vous en éprouvez le besoin, vous pourrez prendre un médicament contre la fièvre et les douleurs comportant du paracétamol selon les recommandations de votre médecin. A chaque fois que vous prendrez un tel médicament, vous le noterez sur le questionnaire dans la partie correspondante.

Vous apporterez ces questionnaires à votre médecin lors de la visite de suivi au 7ème jour.

Au 14ème jour après l'entrée dans l'étude, vous serez contacté par téléphone par une infirmière. Ce rendez vous téléphonique permettra de vérifier la présence ou non de signes/symptômes, et de vous demander comment vous avez supporté les médicaments. A cet effet, nous vous demandons de communiquer à votre médecin les coordonnées téléphoniques de l'endroit où vous serez joignable.

Collection des prélèvements

Une biothèque (conservation d'un échantillon des prélèvements nasaux) va être constituée à partir des prélèvements réalisés à la visite initiale et à J+2. L'ensemble des échantillons permettra d'effectuer des recherches ultérieures sur la grippe. Les échantillons seront congelés et conservés pendant 10 ans au CNR du virus influenzae à Lyon sous la responsabilité de Bruno Lina pour les centres de la région sud et au CNR de Paris sous la responsabilité de Sylvie Van der Werf pour les centres de la région nord.
Cette biothèque ne servira pas à des études génétiques des sujets participant à l'essai.
Votre participation à cette étude ne vous oblige pas à accepter la conservation du prélèvement pour des études ultérieures.

Quels sont les inconvénients possibles ?

L'oseltamivir est un médicament bien toléré. Il peut provoquer cependant des nausées, des vomissements et de la diarrhée, signes qui peuvent être limités par la prise du produit au cours des repas.

Le zanamivir peut provoquer des crises d'asthme chez les asthmatiques ou chez les patients présentant une broncho-pneumopathie chronique obstructive (BPCO). Pour cette raison, vous ne pourrez pas être inclus dans ce protocole si vous présentez un asthme ou une BPCO actuellement en poussée.

Le prélèvement au niveau des narines peut entraîner une gêne passagère sans gravité.

Aucun médicament à l'exception du paracétamol ne doit être pris pendant toute la durée du protocole à votre initiative. La prise d'alcool et les efforts physiques importants ne devront pas être entrepris pendant toute la durée de l'étude.

Les femmes enceintes ne peuvent pas participer à cette étude. Pour cette raison, un test urinaire de grossesse sera réalisé chez les femmes en âge de procréer avant toute inclusion.

Quels sont vos droits ?

A tout moment, vous pouvez décider de quitter cette étude en le disant à votre médecin. Même si vous quittez l'étude, votre médecin vous prescrira le traitement adapté à votre état de santé, en dehors de l'étude.

Votre participation à cette recherche biomédicale n'engendrera pour vous aucun frais supplémentaire par rapport à ceux que vous auriez dans le suivi habituel de cette maladie. Les deux consultations chez votre médecin, la visite de l'infirmière, ainsi que les médicaments seront gratuits pendant la durée de l'étude.

Toutefois, pour pouvoir participer à cette recherche vous devez être affilié(e) ou bénéficier d'un régime de sécurité sociale.

L'Assistance publique - hôpitaux de Paris, qui organise cette recherche biomédicale en qualité de promoteur, a contracté une assurance conformément aux dispositions législatives, garantissant sa responsabilité civile et celle de tout intervenant auprès de la compagnie Gerling France, dont l'adresse est 111 rue de Longchamp, 75116 Paris, par l'intermédiaire de BIOMEDIC INSURE (tel : 02 97 69 19 19) courtier en assurances.

Cette recherche a reçu l'avis favorable du Comité de Protection des Personnes Ile-de-France ……………le / / ainsi que l'autorisation de mise en oeuvre de l'autorité compétente de santé. Il est possible que cette recherche soit interrompue, si les circonstances le nécessitent, par le promoteur ou à la demande de l'autorité de santé.

Dans le cadre de la recherche biomédicale à laquelle l'Assistance publique - hôpitaux de Paris vous propose de participer, un traitement de vos données personnelles va être mis en oeuvre pour permettre d'analyser les résultats de la recherche au regard de l'objectif de cette dernière qui vous a été présenté.
A cette fin, les données médicales vous concernant et les données relatives à vos habitudes de vie seront transmises au promoteur de la recherche ou aux personnes ou sociétés agissant pour son compte, en France ou à l'étranger. Ces données seront identifiées par un numéro de code et vos initiales. Ces données pourront également, dans des conditions assurant leur confidentialité, être transmises aux autorités de santé françaises ou étrangères, à d'autres entités de l'Assistance publique – hôpitaux de Paris. Conformément aux dispositions de la CNIL (loi relative à l'informatique, aux fichiers et aux libertés), vous disposez d'un droit d'accès et de rectification. Vous disposez également d'un droit d'opposition à la transmission des données couvertes par le secret professionnel susceptibles d'être utilisées dans le cadre de cette recherche et d'être traitées.

Vous pouvez également accéder directement ou par l'intermédiaire d'un médecin de votre choix à l'ensemble de vos données médicales en application des dispositions de l'article L. 1111-7 du code de la santé publique. Ces droits s'exercent auprès du médecin qui vous suit dans le cadre de la recherche et qui connaît votre identité.

Votre participation à cette recherche est libre. De plus, vous pouvez exercer à tout moment votre droit de retrait de cette recherche.

Lorsque vous aurez lu cette note d'information et obtenu les réponses aux questions que vous vous posez en interrogeant le médecin, il vous sera proposé, si vous en êtes d'accord, de donner votre consentement écrit en signant le formulaire préparé à cet effet.

Merci pour votre aide et votre participation.

15.4 Consentement de participation

CONSENTEMENT DE PARTICIPATION

Je soussigné(e) M^{e}, Mlle, M. (*nom, prénom*)……………… ………………………….…….……...
accepte librement et volontairement de participer à la recherche biomédicale intitulée «**BIVIR**» dont l'Assistance publique - hôpitaux de Paris est le promoteur et qui m'a été proposée par le Docteur (*nom, prénom*) ……………………………………………., médecin dans cette recherche.

Etant entendu que :

- Le médecin qui m'a informé et a répondu à toutes mes questions, m'a précisé que ma participation est libre et que mon droit de retrait de cette recherche peut s'exercer à tout moment,
- Avant de participer à cette recherche, j'ai bénéficié d'un examen médical dont les résultats m'ont été communiqués,
- Je pourrai avoir communication par le médecin au cours ou à l'issue de la recherche des informations concernant ma santé, qu'il détient,
- J'ai bien compris dans la note d'information qui m'a été remise que pour pouvoir participer à cette recherche je dois être affilié(e) ou bénéficier d'un régime de sécurité sociale. Je confirme que c'est bien le cas,
- Je suis parfaitement conscient(e) que je peux retirer à tout moment mon consentement à ma participation à cette recherche et cela quelles que soient mes raisons et sans supporter aucune responsabilité mais je m'engage dans ce cas à en informer le médecin. Le fait de ne plus participer à cette recherche ne portera pas atteinte à mes relations avec ce médecin,
- Si je le souhaite, à son terme, je serai informé(e) par le médecin des résultats globaux de cette recherche,
- Ma participation à cette recherche implique que je ne pourrai pas participer à une autre recherche biomédicale pendant toute la durée de l'essai (soit 14 jours).
- Mon consentement ne décharge en rien le médecin et le promoteur de l'ensemble de leurs responsabilités et je conserve tous mes droits garantis par la loi.

☐ Je m'oppose à l'utilisation ultérieure des échantillons pour des études ultérieures concernant la grippe.

Fait à
Date : Signature

Signature du médecin qui atteste avoir pleinement expliqué à la personne signataire le but, les modalités ainsi que les risques potentiels de la recherche.

Date : Signature

Les pages de ce document doivent être paraphées par le médecin investigateur et la personne donnant son consentement : la dernière page doit être datée et signée par ces mêmes personnes.
Ce document est à réaliser en 3 exemplaires originaux, dont l'un est conservé 15 ans par le médecin investigateur, un autre remis au patient et le 3ème transmis au promoteur.

16 REFERENCES

Aoki FY, Macleod MD, Paggiaro P, Carewicz O, El Sawy A, Wat C, et al. Early administration of oral oseltamivir increases the benefits of influenza treatment. J Antimicrob Chemother. 2003;51(1):123-9.

Collins PJ, Haire LF, Lin YP, Liu J, Russell RJ, Walker PA, Skehel JJ, Martin SR, Hay AJ, Gamblin SJ. Crystal structures of oseltamivir-resistant influenza virus neuraminidase mutants. Nature. 2008.

Carrat F, Flahault A, Boussard E, Farran N, Dangoumau L, Valleron AJ. Surveillance of influenza-like illness in France. The example of the 1995/1996 epidemic. J Epidemiol Community Health. 1998;52 Suppl 1:32S-38S.

Costagliola D, Flahault A, Galinec D, Garnerin P, Menares J, Valleron AJ. A routine tool for detection and assessment of epidemics of influenza-like syndromes in France. Am J Public Health. 1991;81(1):97-9.

Ferguson NM, Cummings DA, Cauchemez S, Fraser C, Riley S, Meeyai A, Iamsirithaworn S, Burke DS. Strategies for containing an emerging influenza pandemic in Southeast Asia. Nature. 2005 Sep 8;437(7056):209-14.

Ferraris O, Kessler N, Lina B.Sensitivity of influenza viruses to zanamivir and oseltamivir: a study performed on viruses circulating in France prior to the introduction of neuraminidase inhibitors in clinical practice. Antiviral Res. 2005 68:43-8.

Gaglia MA Jr, Cook RL, Kraemer KL, Rothberg MB. Patient knowledge and attitudes about antiviral medication and vaccination for influenza in an internal medicine clinic. Clin Infect Dis. 2007 Nov 1;45(9):1182-8.

Gubareva LV, Kaiser L, Hayden FG. Influenza virus neuraminidase inhibitors. Lancet. 2000;355(9206):827-35.

Gubareva LV, Kaiser L, Matrosovich MN, Soo-Hoo Y, Hayden FG. Selection of influenza virus mutants in experimentally infected volunteers treated with oseltamivir. J Infect Dis. 2001;183(4):523-31.

Hayden FG, Osterhaus AD, Treanor JJ, Fleming DM, Aoki FY, Nicholson KG, et al. Efficacy and safety of the neuraminidase inhibitor zanamivir in the treatment of influenzavirus infections. GG167 Influenza Study Group. N Engl J Med. 1997;337(13):874-80.

Hayden FG, Treanor JJ, Fritz RS, Lobo M, Betts RF, Miller M, et al. Use of the oral neuraminidase inhibitor oseltamivir in experimental human influenza: randomized controlled trials for prevention and treatment. Jama. 1999;282(13):1240-6.

Hayden FG, Belshe R, Villanueva C, Lanno R, Hughes C, Small I, et al. Management of influenza in households: a prospective, randomized comparison of oseltamivir treatment with or without postexposure prophylaxis. J Infect Dis. 2004;189(3):440-9.

de Jong JC, Smith DJ, Lapedes AS, Donatelli I, Campitelli L, Barigazzi G, Van Reeth K, Jones TC, Rimmelzwaan GF, Osterhaus AD, Fouchier RA. Antigenic and genetic evolution of swine influenza A (H3N2) viruses in Europe. J Virol. 2007; 8:4315-22

de Jong MD, Tran TT, Truong HK, Vo MH, Smith GJ, Nguyen VC, Bach VC, Phan TQ, Do QH, Guan Y, Peiris JS, Tran TH, Farrar J.Oseltamivir resistance during treatment of influenza A (H5N1) infection.N Engl J Med. 2005;353:2667-72

Kaiser L, Keene ON, Hammond JM, et al. Impact of zanamivir on antibiotic use for respiratory events following acute influenza in adolescents and adults. Arch Intern Med 2000;160:3234–40.

Kaiser L, Wat C, Mills T, Mahoney P, Ward P, Hayden F. Impact of oseltamivir treatment on influenza-related lower respiratory tract complications and hospitalizations. Arch Intern Med. 2003;163(14):1667-72.

Kiso M, Mitamura K, Sakai-Tagawa Y, Shiraishi K, Kawakami C, Kimura K, Hayden FG, Sugaya N, Kawaoka Y. Resistant influenza A viruses in children treated with oseltamivir: descriptive study. Lancet. 2004 364:759-65.

Lackenby A, Hungnes O, Dudman SG, Meijer A, Paget WJ, Hay AJ, Zambon MC. Emergence of resistance to oseltamivir among influenza A(H1N1) viruses in Europe. Euro Surveill. 2008 Jan 31;13(5). pii: 8026.

Lalezari J, Campion K, KeeneO, Silagy C.Zanamivirforthetreatment of influenza A and B infection in high-risk patients: a pooled analysis of randomized controlled trials. Arch Intern Med 2001;161:212–7.

Lee PY, Matchar DB, Clements DA, et al. Economic analysis of influenza vaccination and antiviral treatment for healthy working adults.Ann Intern Med 2002;137:225–31.

Massarella JW, He GZ, Dorr A, Nieforth K, Ward P, Brown A. The pharmacokinetics and tolerability of the oral neuraminidase inhibitor oseltamivir (Ro 64-0796/GS4104) in healthy adult and elderly volunteers. J Clin Pharmacol. 2000;40(8):836-43.

McKimm-Breschkin JL. Management of influenza virus infections with neuraminidase inhibitors: detection, incidence, and implications of drug resistance. Treat Respir Med. 2005;4:107-16.

The MIST (Management of Influenza in the Southern Hemisphere Trialists) Study Group.Randomised trial of efficacy and safety of inhaled zanamivir in treatment of influenza A and B virus infections. Lancet. 1998;352(9144):1877-81.

Nicholson KG, Aoki FY, Osterhaus AD, Trottier S, Carewicz O, Mercier CH, et al. Efficacy and safety of oseltamivir in treatment of acute influenza: a randomised controlled trial. Neuraminidase Inhibitor Flu Treatment Investigator Group. Lancet. 2000;355(9218):1845-50.

Oo C, Barrett J, Dorr A, Liu B, Ward P. Lack of pharmacokinetic interaction between the oral anti-influenza prodrug oseltamivir and aspirin. Antimicrob Agents Chemother. 2002;46(6):1993-5.

Ortiz JR, Shay DK, Liedtke LA, et al. A national survey of the Infectious Diseases Society of America emerging infections network concerning neuraminidase inhibitor prescription practices and pandemic influenza preparations. Clin Infect Dis 2006;43:494–7.

Puhakka T, Lehti H, Vainionpää R, Jormanainen V, Pulkkinen M, Sharp S, Kerr C, Dempsey M, Ring CJ, Ward C, Tisdale M. Zanamivir: a significant reduction in viral load during treatment in military conscripts with influenza. Scand J Infect Dis. 2003;35(1):52-8.

Rothberg MB, Bellantonio S, Rose DN. Management of influenza in adults older than 65 years of age: cost-effectiveness of rapid testing and antiviral therapy. Ann Intern Med 2003;139:321–9.

Snell P, Oo C, Dorr A, Barrett J. Lack of pharmacokinetic interaction between the oral anti-influenza neuraminidase inhibitor prodrug oseltamivir and antacids. Br J Clin Pharmacol. 2002;54(4):372-7.

Stein J, Louie J,Flanders S, et al. Performance characteristics of clinical diagnosis, a clinical decision rule, and a rapid influenza test in the detection of influenza infection in a community sample of adults. Ann Emerg Med 2005;46:412–9.

Taubenberger JK, Reid AH, Lourens RM, Wang R, Jin G, Fanning TG. Characterization of the 1918 influenza virus polymerase genes. Nature. 2005; 437:889-93.

Treanor JJ, Hayden FG, Vrooman PS, Barbarash R, Bettis R, Riff D, et al. Efficacy and safety of the oral neuraminidase inhibitor oseltamivir in treating acute influenza: a randomized controlled trial. US Oral Neuraminidase Study Group. Jama. 2000;283(8):1016-24.

Welliver R, Monto AS, Carewicz O, Schatteman E, Hassman M, Hedrick J, et al. Effectiveness of oseltamivir in preventing influenza in household contacts: a randomized controlled trial. Jama. 2001;285(6):748-54.

Yen HL, Monto AS, Webster RG, Govorkova EA. Virulence may determine the necessary duration and dosage of oseltamivir treatment for highly pathogenic A/Vietnam/1203/04 influenza virus in mice.J Infect Dis. 2005 192: 665-72.

Zambon M, Hays J, Webster A, Newman R, Keene O. Diagnosis of influenza in the community: relationship of clinical diagnosis to confirmed virological, serologic, or molecular detection of influenza. Arch Intern Med. 2001;161(17):2116-22.
